# National prevalence and trends in food labeling awareness, comprehension, usage, and COVID-19 pandemic-related factors in South Korea, 2014–2022

**DOI:** 10.1038/s41598-024-51948-1

**Published:** 2024-01-31

**Authors:** Yujin Choi, Hyeon Jin Kim, Jaeyu Park, Seung Won Lee, Masoud Rahmati, Ai Koyanagi, Lee Smith, Min Seo Kim, Guillermo F. López Sánchez, Elena Dragioti, Jinseok Lee, Sang Youl Rhee, Sunyoung Kim, Hyunjung Lim, Dong Keon Yon

**Affiliations:** 1 Center for Digital Health, Medical Science Research Institute, Kyung Hee University College of Medicine, Seoul, South Korea; 2 Department of Korean Medicine, Kyung Hee University College of Korean Medicine, Seoul, South Korea; 3 Department of Regulatory Science, Kyung Hee University, Seoul, South Korea; 4 Department of Precision Medicine, Sungkyunkwan University School of Medicine, Suwon, South Korea; 5 Department of Physical Education and Sport Sciences, Faculty of Literature and Human Sciences, Lorestan University, Khoramabad, Iran; 6 Department of Physical Education and Sport Sciences, Faculty of Literature and Humanities, Vali-e-Asr University of Rafsanjan, Rafsanjan, Iran; 7 Research and Development Unit, Parc Sanitari Sant Joan de Deu, Barcelona, Spain; 8 Centre for Health, Performance and Wellbeing, Anglia Ruskin University, Cambridge, UK; 9 Cardiovascular Disease Initiative, Broad Institute of MIT and Harvard, Cambridge, MA USA; 10 Division of Preventive Medicine and Public Health, Department of Public Health Sciences, School of Medicine, University of Murcia, Murcia, Spain; 11 Pain and Rehabilitation Centre, Department of Medical and Health Sciences, Linköping University, Linköping, Sweden; 12 Research Laboratory Psychology of Patients, Families, and Health Professionals, Department of Nursing, School of Health Sciences, University of Ioannina, Ioannina, Greece; 13 Department of Biomedical Engineering, Kyung Hee University, Yongin, South Korea; 14 Department of Endocrinology and Metabolism, Kyung Hee University School of Medicine, Seoul, South Korea; 15 Department of Family Medicine, Kyung Hee University Medical Center, Kyung Hee University College of Medicine, Seoul, South Korea; 16 Department of Medical Nutrition, Graduate School of East-West Medical Science, Kyung Hee University, Yongin, South Korea; 17 Department of Pediatrics, Kyung Hee University College of Medicine, 23 Kyungheedae-Ro, Dongdaemun-Gu, Seoul, 02447 South Korea; 18 Department of Medical Nutrition, Graduate School of East-West Medical Science, Kyung Hee University, 23 Kyungheedae-Ro, Dongdaemun-Gu, Seoul, 02447 South Korea

**Keywords:** Health policy, Nutrition, Public health

## Abstract

Although food labeling on food packages is crucial for promoting a healthy diet, limited research has been conducted on how the COVID-19 pandemic (hereinafter “the pandemic”) has affected food labeling awareness. Therefore, this study aims to analyze the changes in trends in food labeling awareness, comprehension, and usage in South Korea during the pandemic. We utilized a nationwide, large-scale, and long-term dataset provided by the Korea Community Health Survey (KCHS) from 2014 to 2022 (total = 1,756,847 participants). This allowed the researchers to assess the long-term trends in the prevalence of food labeling awareness, comprehension, and usage. Furthermore, we investigated the factors associated with awareness specifically related to the pandemic. In total, 1,756,847 adults (54.19% women) participated in this study. The upward slope in overall food labeling awareness became less pronounced and even exhibited a downward slope during the pandemic (βdiff − 1.759; 95% CI − 1.874 to − 1.644). The upward slope in food labeling comprehension and usage became more pronounced during the pandemic (comprehension: βdiff 0.535; 95% CI 0.436–0.634; usage: βdiff 0.693; 95% CI 0.601–0.785). The vulnerability factors associated with lower food labeling awareness during the pandemic included older age, male, obesity, residing in rural areas, lower household income, lower educational level, smoking, and increased alcohol consumption. This study analyzed the 9-year trend in the prevalence of food labeling awareness, comprehension, and usage based on nationally representative data of adults in South Korea from 2014 to 2022. Our findings suggest that personalized nutrition strategies are needed to recognize vulnerable groups with risk factors and improve food labeling awareness among Korean adults during the pandemic.

## Introduction

The World Health Organization (WHO) has emphasized that a healthy diet assists in preventing many chronic non-communicable diseases such as heart disease, diabetes, and cancer^1^. The WHO recommends that people consume free sugars less than 10% of their total energy intake to prevent diet-related chronic diseases, and those with a total fat energy of at least 20% are consistent with good health^1^. The COVID-19 pandemic (hereinafter “the pandemic”) has significantly altered individuals’ lifestyles worldwide^2–4^, as it has restricted their ability to dine out at restaurants and encouraged them to prepare their meals in their households^2,4–6^.

Concerns about maintaining a healthy diet have increased during the pandemic. For example, those experiencing financial difficulty owing to the pandemic may have no choice in what food to consume, and thus food labeling is irrelevant^2,4,5^. Food labeling for processed food is mandatory currently in South Korea. The labeling includes calories, carbohydrates, sugars, proteins, fats, saturated fats, trans fats, cholesterol, and sodium, and is expressed on the information display surface along with other identified terms, which is mostly the back of the pack^7^. This law for food labeling was enacted in 1996 and has not changed since^7^. While using food labeling on food packages is crucial in maintaining a healthy diet, there is limited research related to the change in food labeling awareness as a result of the pandemic^8–10^. Given this background, the aim of the study was to investigate the long-term trends and prevalence of food labeling awareness, comprehension, and usage before and during the pandemic as well as to determine pandemic-related vulnerability factors of food labeling awareness. The results of the study may assist in determining individual and personalized policies of food labeling to encourage a healthy diet and, ultimately, improve public health.

## Methods

### Study population and data sources

This nationwide, large-scale, long-term study is based on data provided by Korea Community Health Survey (KCHS) from 2014 to 2022^11^. The KCHS was conducted to produce community health statistics for the establishment and evaluation of a community health care plan^12–16^. The participants were recruited based on the method whereby the probability of selection is proportional to the number of households classified by the type of residence. The survey collected a wide range of information, including age, sex, height, weight, region, food labeling awareness, food labeling comprehension, and food labeling usage^12^. Trained interviewers visited the selected households to conduct interviews with household representatives and individuals aged 19 years or older. The data used in this study were approved by the Korea Disease Control and Prevention Agency (KDCA) and Kyung Hee University (KHUH 2022–06-042). This study was conducted in accordance with the principles of the Declaration of Helsinki and all participants signed a written informed consent form^17^.

While 2,061,838 interviewees were conducted in total, participants who did not provide all the necessary information on the covariates used in this study were excluded (excluded n = 304,991). Therefore, 1,756,847 participants were included in the study (men: 804,808 [45.89%]; women: 952,039 [54.19%]).

### Endpoints

Food labeling awareness was determined by the participants’ answers to the question on whether they were aware of food labeling on processed food packaging^12,18^. Participants that answered affirmatively were placed in the “aware group.” Participants in the aware group were further questioned to determine their comprehension of food labeling. Those who replied positively were categorized into the “comprehension group.” The participants in the comprehension group were questioned to determine their food labeling usage. Those who responded positively were categorized into the “use group” (Table S1)^12,18^. Participants who answered 'no' to any one question was considered to have answered 'no' to all subsequent questions.

### Covariate definitions

This study included 9 covariates for consideration; age (19 to 39, 40 to 49, 50 to 59, 60 to 69, and ≥ 70 years), sex, body mass index (BMI), residential areas (urban and rural)^19^, household income (lowest quartile, second quartile, third quartile, and highest quartile), educational level (elementary school or less, middle school, high school, and college or more), smoking status (non-smoker, ex-smoker, and current smoker), alcohol consumption (below a day, once to four days, and five days or more per month), and subjective health level (high, normal, and low). BMI was subdivided into four categories, namely; underweight (< 18.5 kg/m^2^), normal weight (18.5–23 kg/m^2^), overweight (23–25 kg/m^2^), and obese (> 25 kg/m^2^), with respect to the Asia–Pacific BMI^20–24^.

### Statistical analyses

The study used KCHS data from 2014 to 2022 to analyze long-term trends in the prevalence of food labeling awareness, understanding, and use. β-coefficients with 95% confidence intervals (CIs) were calculated using linear regression models for each endpoint before and during the pandemic (2014–2019; 2019–2022), and the differences in β-coefficients were estimated to assess the variations in trends between the before and during pandemic periods^4,25^. All β-coefficients and any differences between them were multiplied by 100 to simplify comprehension. To minimize the impact of potential confounders, all models utilized the following adjusting variables: age group, sex, BMI group, residential area, household income, educational level, smoking status, alcohol consumption, and subjective health level (Supplementary material). Additionally, to address the potential relationship between one or more regressors and the error variance, the Eicker-White heteroskedasticity-consistent estimator was used to estimate standard error values (Tables S2–S4).

$${\text{Endpoint}}={\beta }_{0}+{\beta }_{1}\times year+\sum_{i=2}^{10}{\beta }_{i}\times adjusted\,variable+\epsilon$$

A multivariate logistic regression model was used to analyze the coverage from 2019 to 2022 (2020 versus 2019, 2021 versus 2020, and 2022 versus 2021; Tables S5–7) and to express the ratio of Odds Ratios (OR) with a 95% CIs for identifying factors associated with vulnerability to food labeling awareness during the pandemic^26^. To ensure the representativeness of the study, a survey weighting analysis was conducted using KCHS's unique weighting system, household weighting, for all analyses. This assesses the household extraction rate by incorporating the sample extraction process in sample design, the rate of households suitable for the survey, and the distribution of households based on housing types. The statistical significance was defined as a two-sided p-value less than 0.05. Statistical analyses were performed by utilizing the SAS software (version 9.4; SAS Inc., Cary, NC, USA).

## Results

In total, 1,756,847 adults were included in the KCHS between 2014 and 2022. A large proportion of participants were female (54.19%; n = 952,039; Table 1). Of these groups, 12.2% (n = 214,912) were surveyed in 2014, 12.3% (n = 215,297) in 2015, 12.3% (n = 215,667) in 2016, 12.4% (n = 217,659) in 2017, 10.0% (n = 174,726) in 2018, 10.2% (n = 179,182) in 2019, 10.2% (n = 178,295) in 2020, 10.1% (n = 177,461) in 2021, and 10.5% (n = 183,648) in 2022.

**Table 1:** Baseline characteristics of the participants in the KCHS, 2014–2022 (n = 1,756,847).

Characteristic	2014	2015	2016	2017	2018	2019	2020	2021	2022
Overall (n, %)	214,912 (12.2)	215,297 (12.3)	215,667 (12.3)	217,659 (12.4)	174,726 (10.0)	179,182 (10.2)	178,295 (10.2)	177,461 (10.1)	183,648 (10.5)
Age, year (mean, SD)	50.9 (16.7)	51.7 (16.8)	52.0 (17.0)	52.9 (17.1)	53.4 (17.3)	55.3 (17.7)	54.7 (17.9)	55.2 (17.8)	56.0 (17.7)
Age group, year (n, %)									
19–39	58,702 (27.3)	55,915 (26.0)	55,458 (25.7)	53,105 (24.4)	41,949 (24.0)	38,442 (21.5)	39,952 (22.4)	38,693 (21.8)	37,444 (20.4)
40–49	43,112 (20.1)	40,952 (19.0)	40,011 (18.6)	38,648 (17.8)	29,331 (16.8)	27,271 (15.2)	27,081 (15.2)	26,299 (14.8)	26,276 (14.3)
50–59	44,311 (20.6)	44,359 (20.6)	44,684 (20.7)	44,156 (20.3)	34,836 (19.9)	33,918 (18.9)	33,786 (19.0)	32,846 (18.5)	33,459 (18.2)
60–69	33,803 (15.7)	36,626 (17.0)	37,178 (17.2)	39,507 (18.2)	33,041 (18.9)	35,519 (19.8)	35,554 (19.9)	37,330 (21.0)	40,347 (22.0)
≥ 70	34,984 (16.3)	37,445 (17.4)	38,336 (17.8)	42,243 (19.4)	35,569 (20.4)	44,032 (24.6)	41,922 (23.5)	42,293 (23.8)	46,122 (25.1)
Sex (n, %)									
Male	100,496 (46.8)	100,184 (46.5)	100,219 (46.5)	100,268 (46.1)	80,147 (45.9)	79,655 (44.5)	80,457 (45.1)	80,153 (45.2)	83,229 (45.3)
Female	114,416 (53.2)	115,113 (53.5)	115,448 (53.5)	117,391 (53.9)	94,579 (54.1)	99,527 (55.6)	97,838 (54.9)	97,308 (54.8)	100,419 (54.7)
BMI group (n, %)									
Underweight	11,418 (5.3)	10,981 (5.1)	10,704 (5.0)	10,538 (4.8)	7,112 (4.1)	7,655 (4.3)	7,530 (4.2)	7,955 (4.5)	8,408 (4.6)
Normal weight	97,263 (45.3)	95,196 (44.2)	93,393 (43.3)	93,170 (42.8)	69,954 (40.0)	66,201 (37.0)	73,079 (41.0)	72,734 (41.0)	74,784 (40.7)
Overweight	52,292 (24.3)	53,307 (24.8)	53,023 (24.6)	54,449 (25.0)	42,762 (24.5)	42,523 (23.7)	43,828 (24.6)	43,856 (24.7)	45,328 (24.7)
Obese	53,939 (25.1)	55,813 (25.9)	58,547 (27.2)	59,502 (27.3)	54,898 (31.4)	62,803 (35.1)	53,858 (30.2)	52,916 (29.8)	55,128 (30.0)
Residential areas (n, %)									
Urban	125,792 (58.5)	124,492 (57.8)	124,647 (57.8)	125,089 (57.5)	108,382 (62.0)	106,030 (59.2)	106,273 (59.6)	107,115 (60.4)	111,140 (60.5)
Rural	89,120 (41.5)	90,805 (42.2)	91,020 (42.2)	92,570 (42.5)	66,344 (38.0)	73,152 (40.8)	72,022 (40.4)	70,346 (39.6)	72,508 (39.5)
Household income (n, %)									
Lowest quartile	42,936 (20.0)	42,377 (19.7)	40,340 (18.7)	40,613 (18.7)	24,566 (14.1)	27,939 (15.6)	28,776 (16.1)	27,311 (15.4)	26,257 (14.3)
Second quartile	78,302 (36.4)	77,696 (36.1)	75,587 (35.1)	72,927 (33.5)	54,575 (31.2)	55,760 (31.1)	56,977 (32.0)	55,803 (31.5)	55,072 (30.0)
Third quartile	59,149 (27.5)	60,316 (28.0)	61,238 (28.4)	61,288 (28.2)	47,321 (27.1)	45,609 (25.5)	44,344 (24.9)	43,156 (24.3)	43,927 (23.9)
Highest quartile	34,525 (16.1)	34,908 (16.2)	38,502 (17.9)	42,831 (19.7)	48,264 (27.6)	49,874 (27.8)	48,198 (27.0)	51,191 (28.9)	58,392 (31.8)
Educational level (n, %)									
Elementary school or less	47,321 (22.0)	47,466 (22.1)	47,055 (21.8)	47,390 (21.8)	36,109 (20.7)	42,967 (24.0)	39,048 (21.9)	37,020 (20.9)	37,875 (20.6)
Middle school	24,812 (11.6)	25,083 (11.7)	24,626 (11.4)	25,423 (11.7)	20,278 (11.6)	21,223 (11.8)	20,749 (11.6)	19,932 (11.2)	21,463 (11.7)
High school	64,264 (29.9)	63,832 (29.7)	63,118 (29.3)	63,366 (29.1)	52,876 (30.3)	51,981 (29.0)	53,038 (29.8)	52,949 (29.8)	54,395 (29.6)
College or more	78,515 (36.5)	78,916 (36.7)	80,868 (37.5)	81,480 (37.4)	65,463 (37.5)	63,011 (35.2)	65,460 (36.7)	67,560 (38.1)	69,915 (38.1)
Smoking status (n, %)									
Non-smoker	133,831 (62.3)	134,497 (62.5)	133,986 (62.1)	137,735 (63.3)	111,065 (63.6)	113,545 (63.4)	116,956 (65.6)	115,534 (65.1)	113,843 (62.0)
Ex-smoker	36,326 (16.9)	40,278 (18.7)	40,741 (18.9)	40,875 (18.8)	32,224 (18.4)	35,978 (20.1)	32,250 (18.1)	33,425 (18.8)	39,729 (21.6)
Current smoker	44,755 (20.8)	40,522 (18.8)	40,940 (19.0)	39,049 (17.9)	31,437 (18.0)	29,659 (16.6)	29,089 (16.3)	28,502 (16.1)	30,076 (16.4)
Alcohol consumption, days/month (n, %)									
< 1	98,237 (45.7)	98,688 (45.8)	100,693 (46.7)	102,442 (47.1)	83,473 (47.8)	91,478 (51.1)	99,316 (55.7)	101,175 (57.0)	98,321 (53.5)
1–4	67,464 (31.4)	67,220 (31.2)	67,152 (31.1)	66,160 (30.4)	52,176 (29.9)	51,330 (28.7)	47,765 (26.8)	45,874 (25.9)	51,753 (28.2)
≥ 5	49,211 (22.9)	49,389 (22.9)	47,822 (22.2)	49,057 (22.5)	39,077 (22.4)	36,374 (20.3)	31,214 (17.5)	30,412 (17.1)	33,574 (18.3)
Subjective health level (n, %)									
High	80,728 (37.6)	82,211 (38.2)	80,765 (37.5)	81,293 (37.4)	62,879 (36.0)	60,433 (33.7)	85,068 (47.7)	71,291 (40.2)	73,060 (39.8)
Normal	91,878 (42.8)	90,796 (42.2)	92,733 (43.0)	92,552 (42.5)	78,641 (45.0)	80,500 (44.9)	68,840 (38.6)	75,402 (42.5)	75,608 (41.2)
Low	42,306 (19.7)	42,290 (19.6)	42,169 (19.6)	43,814 (20.1)	33,206 (19.0)	38,249 (21.4)	24,387 (13.7)	30,768 (17.3)	34,980 (19.1)

*BMI* body mass index, *KCHS* Korea Community Health Survey, *SD* standard deviation.

**Figure 1:**
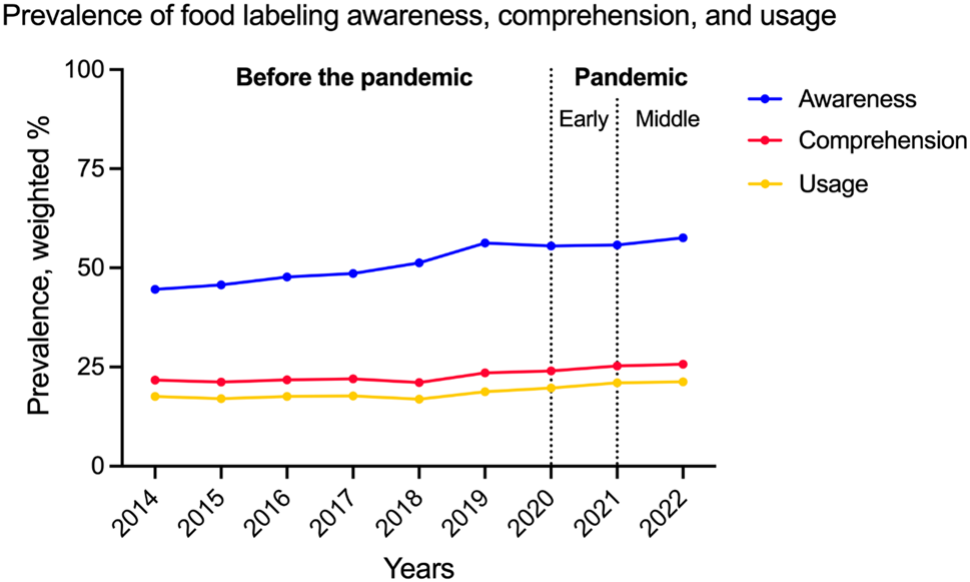
Prevalence of food labeling awareness, comprehension, and usage in the KCHS, 2014–2022 (n = 1,756,847).

Tables 2, 3, and 4 present the changes in trends regarding the proportion of individuals who reported awareness of the presence of food labeling on the products they buy, those who read and comprehend the labeling on the food, and those who make use of the food labels when buying products, respectively. All trends exhibited a constant rate of growth in awareness, comprehension, and utilization of food labeling (Fig. 1).

**Table 2:** Prevalence of food labeling awareness in the KCHS, 2014–2022 (n = 1,756,847).

Characteristic	2014	2015	2016	2017	2018	2019	2020	2021	2022	Before the pandemic, β (2014–2019)	After the pandemic, β (2019–2022)	Trend difference, βdiff
Overall weighted % (95% CI)	44.6 (44.3 to 44.8)	45.7 (45.5 to 46.0)	47.7 (47.4 to 47.9)	48.6 (48.4 to 48.8)	51.3 (51.1 to 51.6)	56.3 (56.1 to 56.6)	55.5 (55.3 to 55.8)	55.8 (55.5 to 56.0)	57.6 (57.3 to 57.8)	**2.158 (2.105 to 2.210)**	**0.399 (0.297 to 0.501)**	**− 1.759 (− 1.874 to − 1.644)**
Age group, year weighted % (95% CI)												
19–39	64.3 (63.9 to 64.6)	64.4 (64.0 to 64.8)	66.9 (66.5 to 67.3)	68.9 (68.5 to 69.3)	70.4 (70.0 to 70.8)	75.6 (75.2 to 76.0)	74.2 (73.8 to 74.6)	73.4 (73.0 to 73.9)	75.8 (75.4 to 76.2)	**2.121 (2.023 to 2.219)**	**− **0.029 (**− **0.222 to 0.165)	**− 2.149 (− 2.366 to − 1.933)**
40–49	58.8 (58.4 to 59.3)	60.0 (59.6 to 60.5)	63.0 (62.5 to 63.5)	65.3 (64.8 to 65.8)	66.3 (65.7 to 66.8)	74.2 (73.7 to 74.7)	71.9 (71.4 to 72.4)	71.6 (71.1 to 72.1)	72.9 (72.4 to 73.5)	**2.712 (2.593 to 2.831)**	**− 0.424 (− 0.661 to − 0.187)**	**− 3.136 (− 3.401 to − 2.871)**
50–59	44.4 (43.9 to 44.9)	47.5 (47.0 to 47.9)	50.4 (50.0 to 50.9)	52.5 (52.0 to 52.9)	56.4 (55.9 to 56.9)	65.6 (65.1 to 66.1)	64.1 (63.6 to 64.7)	64.4 (63.9 to 65.0)	67.2 (66.7 to 67.7)	**3.781 (3.665 to 3.897)**	**0.497 (0.271 to 0.723)**	**− 3.284 (− 3.538 to − 3.030)**
60–69	27.1 (26.7 to 27.6)	32.0 (31.5 to 32.5)	33.7 (33.3 to 34.2)	36.3 (35.8 to 36.7)	42.3 (41.8 to 42.9)	51.7 (51.2 to 52.3)	50.7 (50.2 to 51.2)	52.7 (52.2 to 53.2)	56.1 (55.6 to 56.6)	**4.480 (4.360 to 4.599)**	**1.549 (1.324 to 1.774)**	**− 2.930 (− 3.185 to − 2.676)**
≥ 70	11.0 (10.6 to 11.3)	13.6 (13.3 to 14.0)	14.0 (13.7 to 14.4)	15.5 (15.1 to 15.8)	19.9 (19.5 to 20.3)	25.0 (24.6 to 25.4)	24.3 (23.9 to 24.7)	25.8 (25.3 to 26.2)	28.3 (27.9 to 28.7)	**2.633 (2.544 to 2.723)**	**1.147 (0.964 to 1.330)**	**− 1.487 (− 1.690 to − 1.283)**
Sex weighted % (95% CI)												
Male	36.8 (36.5 to 37.1)	37.9 (37.6 to 38.2)	40.1 (39.8 to 40.4)	41.1 (40.8 to 41.4)	43.8 (43.5 to 44.2)	51.6 (51.3 to 52.0)	50.6 (50.3 to 51.0)	50.4 (50.1 to 50.8)	52.5 (52.2 to 52.9)	**2.585 (2.508 to 2.661)**	**0.264 (0.111 to 0.418)**	**− 2.320 (− 2.492 to − 2.149)**
Female	51.3 (51.1 to 51.6)	52.5 (52.2 to 52.8)	54.2 (53.9 to 54.5)	55.1 (54.8 to 55.3)	57.7 (57.4 to 58.0)	60.1 (59.8 to 60.4)	59.6 (59.3 to 59.9)	60.2 (59.9 to 60.5)	61.7 (61.4 to 62.0)	**1.701 (1.630 to 1.772)**	**0.552 (0.417 to 0.688)**	**− 1.149 (− 1.302 to − 0.996)**
BMI group weighted % (95% CI)												
Underweight	46.0 (45.0 to 46.9)	45.9 (45.0 to 46.9)	46.3 (45.3 to 47.2)	47.2 (46.3 to 48.2)	50.9 (49.8 to 52.1)	55.9 (54.8 to 57.0)	51.3 (50.2 to 52.4)	51.7 (50.6 to 52.8)	53.7 (52.7 to 54.8)	**1.754 (1.511 to 1.997)**	**− 0.577 (− 1.066 to − 0.089)**	**− 2.332 (− 2.877 to − 1.786)**
Normal weight	47.1 (46.8 to 47.4)	48.3 (48.0 to 48.6)	49.8 (49.5 to 50.1)	50.9 (50.5 to 51.2)	53.8 (53.5 to 54.2)	58.9 (58.5 to 59.3)	56.6 (56.2 to 56.9)	56.5 (56.1 to 56.9)	58.2 (57.9 to 58.6)	**2.106 (2.024 to 2.188)**	**− 0.168 (− 0.331 to − 0.005)**	**− 2.274 (− 2.456 to − 2.092)**
Overweight	42.2 (41.8 to 42.7)	43.4 (43.0 to 43.8)	45.7 (45.3 to 46.1)	46.4 (46.0 to 46.8)	49.4 (49.0 to 49.9)	55.3 (54.8 to 55.7)	54.3 (53.8 to 54.8)	54.6 (54.2 to 55.1)	56.3 (55.9 to 56.8)	**2.352 (2.245 to 2.459)**	**0.363 (0.155 to 0.570)**	**− 1.989 (− 2.223 to − 1.755)**
Obese	41.9 (41.5 to 42.3)	43.6 (43.1 to 44.0)	46.2 (45.8 to 46.6)	47.4 (47.0 to 47.8)	49.6 (49.2 to 50.1)	54.4 (54.0 to 54.8)	55.7 (55.3 to 56.1)	56.3 (55.9 to 56.8)	58.3 (57.9 to 58.7)	**2.364 (2.267 to 2.461)**	**1.220 (1.040 to 1.400)**	**− 1.145 (− 1.349 to − 0.940)**
Residential areas weighted % (95% CI)												
Urban	50.7 (50.5 to 51.0)	51.5 (51.2 to 51.7)	53.5 (53.2 to 53.8)	55.3 (55.1 to 55.6)	55.9 (55.6 to 56.2)	62.4 (62.1 to 62.6)	60.8 (60.5 to 61.1)	60.9 (60.6 to 61.2)	62.4 (62.1 to 62.7)	**2.066 (1.998 to 2.134)**	0.027 (**− **0.102 to 0.156)	**− 2.039 (− 2.185 to − 1.893)**
Rural	35.8 (35.5 to 36.1)	37.9 (37.6 to 38.2)	39.6 (39.3 to 39.9)	39.6 (39.2 to 39.9)	43.8 (43.4 to 44.2)	47.6 (47.3 to 48.0)	47.8 (47.4 to 48.1)	48.0 (47.6 to 48.3)	50.2 (49.8 to 50.6)	**2.146 (2.065 to 2.227)**	**0.796 (0.633 to 0.958)**	**− 1.350 (− 1.532 to − 1.169)**
Household income weighted % (95% CI)												
Lowest quartile	20.1 (19.7 to 20.5)	21.9 (21.5 to 22.3)	22.4 (22.0 to 22.8)	22.2 (21.8 to 22.6)	24.2 (23.7 to 24.8)	27.0 (26.4 to 27.5)	28.4 (27.9 to 28.9)	29.7 (29.2 to 30.2)	28.8 (28.3 to 29.4)	**1.136 (1.029 to 1.243)**	**0.694 (0.455 to 0.933)**	**− 0.442 (− 0.704 to − 0.180)**
Second quartile	43.5 (43.1 to 43.8)	43.6 (43.3 to 44.0)	45.5 (45.1 to 45.9)	45.1 (44.8 to 45.5)	45.1 (44.7 to 45.5)	50.9 (50.5 to 51.3)	50.9 (50.5 to 51.3)	50.5 (50.1 to 50.9)	51.4 (51.0 to 51.8)	**1.139 (1.048 to 1.230)**	0.123 (**− **0.063 to 0.309)	**− 1.016 (− 1.223 to − 0.809)**
Third quartile	55.7 (55.3 to 56.1)	56.5 (56.1 to 56.9)	57.6 (57.2 to 58.0)	59.0 (58.6 to 59.4)	59.7 (59.2 to 60.1)	65.4 (65.0 to 65.8)	64.3 (63.9 to 64.8)	63.9 (63.5 to 64.4)	65.0 (64.6 to 65.5)	**1.648 (1.548 to 1.748)**	**− **0.158 (**− **0.356 to 0.040)	**− 1.806 (− 2.027 to − 1.584)**
Highest quartile	58.4 (57.9 to 58.9)	60.8 (60.3 to 61.3)	62.6 (62.1 to 63.1)	64.8 (64.3 to 65.2)	64.0 (63.5 to 64.4)	70.6 (70.2 to 71.0)	69.1 (68.7 to 69.6)	68.6 (68.2 to 69.0)	70.7 (70.3 to 71.0)	**2.106 (1.995 to 2.216)**	0.008 (**− **0.165 to 0.181)	**− 2.097 (− 2.303 to − 1.892)**
Educational level weighted % (95% CI)												
Elementary school or less	13.2 (12.9 to 13.5)	15.4 (15.1 to 15.8)	16.4 (16.1 to 16.8)	16.7 (16.4 to 17.0)	20.8 (20.4 to 21.2)	25.1 (24.7 to 25.5)	24.0 (23.6 to 24.4)	24.8 (24.3 to 25.2)	25.5 (25.0 to 25.9)	**2.149 (2.062 to 2.235)**	0.178 (**− **0.012 to 0.368)	**− 1.970 (− 2.179 to − 1.761)**
Middle school	31.1 (30.5 to 31.7)	33.1 (32.5 to 33.7)	34.7 (34.1 to 35.3)	35.1 (34.5 to 35.7)	39.6 (38.9 to 40.2)	47.4 (46.7 to 48.1)	45.5 (44.8 to 46.2)	45.2 (44.5 to 45.9)	47.6 (46.9 to 48.2)	**2.841 (2.692 to 2.991)**	0.021 (**− **0.279 to 0.321)	**− 2.820 (− 3.156 to − 2.485)**
High school	49.3 (48.9 to 49.7)	49.9 (49.5 to 50.3)	52.1 (51.8 to 52.5)	53.3 (52.9 to 53.7)	55.4 (55.0 to 55.9)	63.6 (63.2 to 64.0)	61.2 (60.8 to 61.6)	60.5 (60.1 to 60.9)	62.8 (62.4 to 63.2)	**2.484 (2.388 to 2.580)**	**− 0.286 (− 0.470 to − 0.102)**	**− 2.770 (− 2.977 to − 2.563)**
College or more	63.8 (63.5 to 64.1)	64.6 (64.3 to 64.9)	66.3 (65.9 to 66.6)	67.8 (67.5 to 68.1)	68.5 (68.1 to 68.8)	74.7 (74.3 to 75.0)	72.9 (72.6 to 73.3)	72.2 (71.9 to 72.5)	73.9 (73.6 to 74.2)	**1.887 (1.806 to 1.968)**	**− 0.269 (− 0.418 to − 0.120)**	**− 2.156 (− 2.326 to − 1.986)**
Smoking status weighted % (95% CI)												
Non-smoker	49.9 (49.7 to 50.2)	51.4 (51.1 to 51.6)	53.1 (52.8 to 53.4)	53.8 (53.5 to 54.0)	56.2 (55.9 to 56.5)	59.6 (59.3 to 59.9)	58.3 (58.1 to 58.6)	59.0 (58.7 to 59.3)	60.7 (60.4 to 61.0)	**1.791 (1.724 to 1.857)**	**0.410 (0.282 to 0.537)**	**− 1.381 (− 1.524 to − 1.237)**
Ex-smoker	32.8 (32.3 to 33.3)	34.1 (33.7 to 34.6)	35.7 (35.2 to 36.1)	36.9 (36.4 to 37.4)	39.4 (38.8 to 39.9)	48.4 (47.9 to 48.9)	47.9 (47.4 to 48.5)	47.8 (47.3 to 48.4)	51.0 (50.6 to 51.5)	**2.726 (2.607 to 2.845)**	**0.820 (0.593 to 1.047)**	**− 1.906 (− 2.163 to − 1.650)**
Current smoker	38.0 (37.6 to 38.5)	38.6 (38.1 to 39.1)	41.8 (41.3 to 42.3)	42.8 (42.3 to 43.3)	46.4 (45.9 to 47.0)	53.5 (53.0 to 54.1)	52.7 (52.1 to 53.2)	52.1 (51.5 to 52.6)	54.2 (53.7 to 54.8)	**2.802 (2.681 to 2.923)**	0.153 (**− **0.100 to 0.406)	**− 2.649 (− 2.930 to − 2.368)**
Alcohol consumption, days/month weighted % (95% CI)												
< 1	40.7 (40.4 to 41.0)	42.2 (41.9 to 42.5)	43.3 (43.0 to 43.6)	43.6 (43.3 to 43.9)	46.9 (46.5 to 47.2)	50.9 (50.6 to 51.3)	51.3 (51.0 to 51.6)	51.9 (51.6 to 52.2)	52.8 (52.5 to 53.1)	**1.855 (1.779 to 1.931)**	**0.626 (0.484 to 0.768)**	**− 1.229 (− 1.390 to − 1.068)**
1–4	53.9 (53.5 to 54.2)	54.6 (54.2 to 55.0)	57.0 (56.7 to 57.4)	58.9 (58.5 to 59.2)	60.2 (59.7 to 60.6)	66.5 (66.1 to 66.9)	65.1 (64.7 to 65.5)	65.2 (64.7 to 65.6)	67.3 (66.9 to 67.7)	**2.295 (2.200 to 2.389)**	**0.254 (0.072 to 0.437)**	**− 2.040 (− 2.246 to − 1.835)**
≥ 5	39.5 (39.0 to 39.9)	40.8 (40.4 to 41.3)	43.6 (43.1 to 44.0)	45.4 (44.9 to 45.8)	49.0 (48.6 to 49.5)	55.5 (55.0 to 56.1)	54.2 (53.7 to 54.8)	54.5 (54.0 to 55.1)	56.4 (55.9 to 56.9)	**2.977 (2.866 to 3.089)**	**0.269 (0.035 to 0.503)**	**− 2.708 (− 2.968 to − 2.449)**
Subjective health level weighted % (95% CI)												
High	53.6 (53.3 to 53.9)	54.5 (54.1 to 54.8)	56.5 (56.2 to 56.9)	57.8 (57.4 to 58.1)	58.3 (57.9 to 58.7)	65.4 (65.0 to 65.8)	62.6 (62.3 to 63.0)	63.1 (62.7 to 63.4)	66.2 (65.9 to 66.6)	**1.992 (1.906 to 2.079)**	**0.423 (0.263 to 0.583)**	**− 1.569 (− 1.751 to − 1.387)**
Normal	46.0 (45.7 to 46.4)	47.1 (46.8 to 47.4)	49.3 (48.9 to 49.6)	50.5 (50.2 to 50.8)	53.2 (52.9 to 53.6)	58.9 (58.6 to 59.3)	54.5 (54.1 to 54.9)	56.9 (56.5 to 57.2)	59.1 (58.8 to 59.5)	**2.378 (2.299 to 2.458)**	**0.233 (0.078 to 0.388)**	**− 2.145 (− 2.319 to − 1.971)**
Low	24.1 (23.7 to 24.5)	25.9 (25.5 to 26.3)	27.2 (26.7 to 27.6)	27.7 (27.2 to 28.1)	33.7 (33.1 to 34.2)	36.6 (36.1 to 37.1)	33.6 (33.0 to 34.2)	36.2 (35.7 to 36.8)	36.1 (35.6 to 36.6)	**2.428 (2.320 to 2.535)**	0.008 (**− **0.215 to 0.231)	**− 2.420 (− 2.668 to − 2.173)**

*BMI* body mass index, *CI* confidence interval, *KCHS* Korea Community Health Survey.

The beta values were multiplied by 100 as a result of their minimal number. Numbers in bold indicate a significant difference (P < 0.05).

*The model was adjusted for age (19 to 39, 40 to 49, 50 to 59, 60 to 69, and ≥ 70 years), sex, body mass index (BMI; underweight, normal weight, overweight, and obese), residential areas (urban and rural), household income (lowest quartile, second quartile, third quartile, and highest quartile), educational level (elementary school or less, middle school, high school, and college or more), smoking status (non-smoker, ex-smoker, and current smoker), alcohol consumption (below a day, once to four days, and five days or more per month), and subjective health level (high, normal, and low).

**Table 3:** Prevalence of food labeling comprehension in the KCHS, 2014–2022 (n = 1,756,847).

Characteristic	2014	2015	2016	2017	2018	2019	2020	2021	2022	before the pandemic, β (2014–2019)	after the pandemic, β (2019–2022)	Trend difference, βdiff
Overall weighted % (95% CI)	21.7 (21.5 to 21.9)	21.2 (21.0 to 21.3)	21.8 (21.6 to 22.0)	22.0 (21.9 to 22.2)	21.1 (20.9 to 21.3)	23.5 (23.3 to 23.7)	24.0 (23.8 to 24.2)	25.3 (25.1 to 25.5)	25.7 (25.5 to 25.9)	**0.259 (0.215 to 0.303)**	**0.794 (0.705 to 0.882)**	**0.535 (0.436 to 0.634)**
Age group, year weighted % (95% CI)												
19–39	35.3 (34.9 to 35.7)	33.9 (33.5 to 34.2)	35.2 (34.8 to 35.6)	35.5 (35.1 to 35.9)	33.8 (33.3 to 34.2)	36.9 (36.4 to 37.4)	37.3 (36.8 to 37.8)	37.6 (37.1 to 38.1)	38.8 (38.3 to 39.3)	**0.193 (0.091 to 0.296)**	**0.603 (0.385 to 0.820)**	**0.410 (0.169 to 0.650)**
40–49	30.0 (29.6 to 30.4)	29.9 (29.5 to 30.4)	31.0 (30.5 to 31.4)	32.8 (32.3 to 33.3)	30.3 (29.8 to 30.8)	35.2 (34.6 to 35.8)	35.2 (34.6 to 35.8)	36.1 (35.6 to 36.7)	36.5 (35.9 to 37.1)	**0.803 (0.685 to 0.920)**	**0.474 (0.218 to 0.731)**	**-0.328 (-0.611 to -0.046)**
50–59	19.1 (18.8 to 19.5)	19.9 (19.5 to 20.3)	20.8 (20.4 to 21.2)	22.1 (21.7 to 22.5)	21.6 (21.1 to 22.0)	26.5 (26.1 to 27.0)	27.1 (26.6 to 27.6)	29.5 (29.0 to 30.0)	30.1 (29.6 to 30.6)	**1.212 (1.113 to 1.310)**	**1.305 (1.090 to 1.520)**	0.093 (-0.143 to 0.330)
60–69	9.7 (9.4 to 10.0)	11.1 (10.8 to 11.4)	11.6 (11.2 to 11.9)	12.5 (12.2 to 12.8)	13.8 (13.4 to 14.2)	18.0 (17.6 to 18.4)	18.3 (17.9 to 18.7)	20.9 (20.5 to 21.3)	22.5 (22.1 to 22.9)	**1.442 (1.356 to 1.527)**	**1.627 (1.446 to 1.808)**	0.185 (-0.015 to 0.385)
≥ 70	3.3 (3.1 to 3.5)	4.0 (3.8 to 4.2)	4.0 (3.8 to 4.2)	4.1 (3.9 to 4.3)	4.9 (4.7 to 5.1)	6.8 (6.5 to 7.0)	6.6 (6.4 to 6.8)	8.0 (7.7 to 8.2)	8.7 (8.5 to 9.0)	**0.597 (0.546 to 0.649)**	**0.721 (0.611 to 0.831)**	**0.124 (0.002 to 0.245)**
Sex weighted % (95% CI)												
Male	12.4 (12.1 to 12.6)	12.1 (11.9 to 12.3)	13.0 (12.8 to 13.2)	13.1 (12.9 to 13.3)	12.5 (12.2 to 12.7)	15.3 (15.0 to 15.5)	16.4 (16.2 to 16.7)	17.6 (17.3 to 17.8)	18.1 (17.9 to 18.4)	**0.437 (0.384 to 0.490)**	**0.964 (0.850 to 1.079)**	**0.527 (0.401 to 0.654)**
Female	29.9 (29.6 to 30.1)	29.0 (28.8 to 29.3)	29.4 (29.2 to 29.7)	29.6 (29.4 to 29.9)	28.4 (28.1 to 28.7)	30.1 (29.8 to 30.4)	30.3 (30.0 to 30.6)	31.7 (31.4 to 32.0)	32.1 (31.8 to 32.4)	-0.010 (-0.076 to 0.055)	**0.722 (0.594 to 0.851)**	**0.732 (0.588 to 0.877)**
BMI group weighted % (95% CI)												
Underweight	25.7 (24.9 to 26.5)	24.7 (23.8 to 25.5)	24.3 (23.5 to 25.1)	24.7 (23.9 to 25.5)	23.9 (22.9 to 24.9)	26.9 (25.9 to 27.9)	24.9 (23.9 to 25.9)	26.7 (25.8 to 27.7)	26.7 (25.7 to 27.6)	0.076 (-0.137 to 0.290)	0.107 (-0.327 to 0.541)	0.031 (-0.453 to 0.514)
Normal weight	24.5 (24.2 to 24.8)	23.9 (23.6 to 24.1)	24.4 (24.2 to 24.7)	24.9 (24.6 to 25.2)	23.8 (23.5 to 24.1)	26.7 (26.4 to 27.1)	26.3 (26.0 to 26.7)	27.2 (26.9 to 27.6)	27.8 (27.4 to 28.1)	**0.300 (0.229 to 0.372)**	**0.405 (0.258 to 0.552)**	0.104 (-0.059 to 0.268)
Overweight	19.1 (18.8 to 19.5)	18.6 (18.2 to 18.9)	19.4 (19.0 to 19.7)	19.4 (19.1 to 19.8)	18.9 (18.5 to 19.2)	21.3 (20.9 to 21.7)	22.1 (21.7 to 22.5)	23.5 (23.1 to 23.9)	23.9 (23.5 to 24.3)	**0.333 (0.247 to 0.419)**	**0.910 (0.735 to 1.085)**	**0.577 (0.383 to 0.772)**
Obese	18.2 (17.9 to 18.5)	18.4 (18.0 to 18.7)	19.3 (19.0 to 19.6)	19.4 (19.1 to 19.8)	19.0 (18.7 to 19.3)	21.2 (20.8 to 21.5)	22.4 (22.0 to 22.7)	23.9 (23.5 to 24.3)	24.4 (24.0 to 24.7)	**0.498 (0.421 to 0.575)**	**1.118 (0.966 to 1.270)**	**0.620 (0.449 to 0.791)**
Residential areas weighted % (95% CI)												
Urban	25.6 (25.4 to 25.9)	25.2 (24.9 to 25.4)	25.8 (25.6 to 26.1)	26.3 (26.1 to 26.6)	24.5 (24.2 to 24.7)	27.6 (27.4 to 27.9)	28.0 (27.7 to 28.2)	29.0 (28.8 to 29.3)	29.3 (29.0 to 29.5)	**0.233 (0.172 to 0.293)**	**0.593 (0.473 to 0.713)**	**0.360 (0.226 to 0.495)**
Rural	16.1 (15.8 to 16.3)	15.7 (15.5 to 15.9)	16.3 (16.0 to 16.5)	16.2 (16.0 to 16.5)	15.6 (15.3 to 15.9)	17.5 (17.3 to 17.8)	18.2 (18.0 to 18.5)	19.6 (19.3 to 19.9)	20.4 (20.1 to 20.6)	**0.199 (0.138 to 0.260)**	**0.979 (0.852 to 1.107)**	**0.780 (0.639 to 0.922)**
Household income weighted % (95% CI)												
Lowest quartile	8.1 (7.9 to 8.4)	8.2 (8.0 to 8.5)	8.3 (8.1 to 8.6)	8.0 (7.7 to 8.3)	7.5 (7.2 to 7.8)	9.0 (8.7 to 9.3)	9.9 (9.6 to 10.3)	11.3 (10.9 to 11.6)	10.3 (9.9 to 10.6)	0.048 (-0.022 to 0.118)	**0.529 (0.371 to 0.687)**	**0.481 (0.308 to 0.654)**
Second quartile	20.4 (20.1 to 20.7)	19.3 (19.0 to 19.5)	19.8 (19.5 to 20.1)	19.0 (18.7 to 19.3)	16.7 (16.3 to 17.0)	19.3 (19.0 to 19.7)	20.3 (20.0 to 20.6)	21.3 (21.0 to 21.7)	21.4 (21.1 to 21.8)	**-0.396 (-0.468 to -0.324)**	**0.730 (0.580 to 0.880)**	**1.126 (0.960 to 1.292)**
Third quartile	27.8 (27.5 to 28.2)	27.0 (26.6 to 27.3)	27.4 (27.0 to 27.7)	27.7 (27.4 to 28.1)	25.4 (25.0 to 25.8)	28.0 (27.6 to 28.5)	28.7 (28.3 to 29.1)	29.9 (29.4 to 30.3)	29.8 (29.4 to 30.2)	**-0.104 (-0.195 to -0.012)**	**0.653 (0.465 to 0.841)**	**0.757 (0.548 to 0.966)**
Highest quartile	31.0 (30.5 to 31.4)	31.0 (30.5 to 31.5)	31.0 (30.5 to 31.4)	32.3 (31.9 to 32.8)	28.8 (28.4 to 29.2)	32.2 (31.8 to 32.6)	32.6 (32.2 to 33.0)	33.2 (32.8 to 33.7)	33.7 (33.3 to 34.1)	0.028 (-0.079 to 0.135)	**0.520 (0.342 to 0.698)**	**0.492 (0.284 to 0.700)**
Educational level weighted % (95% CI)												
Elementary school or less	3.9 (3.7 to 4.1)	4.2 (4.1 to 4.4)	4.2 (4.0 to 4.4)	4.3 (4.1 to 4.5)	4.8 (4.6 to 5.0)	6.5 (6.2 to 6.7)	5.8 (5.6 to 6.1)	6.7 (6.5 to 7.0)	6.7 (6.5 to 7.0)	**0.417 (0.368 to 0.466)**	**0.158 (0.049 to 0.266)**	**-0.259 (-0.378 to -0.140)**
Middle school	11.2 (10.8 to 11.6)	11.4 (11.0 to 11.8)	12.2 (11.8 to 12.6)	11.9 (11.5 to 12.3)	12.2 (11.7 to 12.6)	15.6 (15.1 to 16.1)	15.3 (14.8 to 15.8)	16.3 (15.8 to 16.8)	17.5 (17.0 to 18.0)	**0.665 (0.561 to 0.769)**	**0.669 (0.446 to 0.892)**	0.004 (-0.242 to 0.250)
High school	22.7 (22.4 to 23.0)	22.2 (21.8 to 22.5)	22.6 (22.3 to 22.9)	22.8 (22.5 to 23.2)	21.5 (21.1 to 21.8)	25.5 (25.1 to 25.9)	25.2 (24.8 to 25.6)	26.5 (26.1 to 26.8)	27.0 (26.6 to 27.4)	**0.325 (0.242 to 0.407)**	**0.585 (0.417 to 0.752)**	**0.260 (0.073 to 0.446)**
College or more	34.8 (34.5 to 35.2)	33.6 (33.3 to 34.0)	34.3 (34.0 to 34.7)	34.9 (34.5 to 35.2)	32.5 (32.2 to 32.9)	36.2 (35.8 to 36.6)	36.7 (36.4 to 37.1)	37.2 (36.8 to 37.6)	37.6 (37.3 to 38.0)	**0.098 (0.014 to 0.182)**	**0.474 (0.310 to 0.638)**	**0.376 (0.192 to 0.560)**
Smoking status weighted % (95% CI)												
Non-smoker	27.6 (27.4 to 27.9)	27.0 (26.7 to 27.2)	27.5 (27.3 to 27.8)	27.5 (27.3 to 27.8)	26.2 (26.0 to 26.5)	28.4 (28.2 to 28.7)	28.2 (27.9 to 28.5)	29.7 (29.4 to 30.0)	30.4 (30.2 to 30.7)	0.048 (-0.011 to 0.108)	**0.750 (0.631 to 0.868)**	**0.701 (0.569 to 0.834)**
Ex-smoker	11.0 (10.7 to 11.4)	11.1 (10.8 to 11.4)	11.7 (11.4 to 12.0)	11.8 (11.5 to 12.1)	11.2 (10.9 to 11.6)	14.8 (14.4 to 15.1)	15.7 (15.3 to 16.1)	16.7 (16.3 to 17.1)	18.1 (17.8 to 18.5)	**0.559 (0.478 to 0.640)**	**1.113 (0.945 to 1.281)**	**0.554 (0.367 to 0.740)**
Current smoker	12.5 (12.2 to 12.8)	11.8 (11.5 to 12.2)	13.0 (12.7 to 13.4)	13.3 (13.0 to 13.7)	13.0 (12.6 to 13.4)	15.2 (14.8 to 15.6)	16.6 (16.2 to 17.0)	17.5 (17.1 to 17.9)	18.1 (17.6 to 18.5)	**0.480 (0.396 to 0.564)**	**0.935 (0.746 to 1.124)**	**0.455 (0.248 to 0.662)**
Alcohol consumption, days/month weighted % (95% CI)												
< 1	21.6 (21.3 to 21.8)	21.1 (20.9 to 21.4)	21.3 (21.0 to 21.5)	20.9 (20.7 to 21.2)	20.6 (20.3 to 20.8)	22.2 (22.0 to 22.5)	23.1 (22.8 to 23.3)	24.7 (24.4 to 24.9)	24.7 (24.4 to 25.0)	0.038 (-0.025 to 0.101)	**0.906 (0.785 to 1.026)**	**0.868 (0.732 to 1.004)**
1–4	27.0 (26.6 to 27.3)	26.0 (25.7 to 26.4)	27.0 (26.7 to 27.4)	27.7 (27.3 to 28.0)	25.6 (25.2 to 26.0)	29.0 (28.7 to 29.4)	29.2 (28.7 to 29.6)	30.0 (29.6 to 30.5)	31.2 (30.8 to 31.6)	**0.275 (0.189 to 0.361)**	**0.731 (0.553 to 0.909)**	**0.456 (0.259 to 0.654)**
≥ 5	14.7 (14.3 to 15.0)	14.7 (14.3 to 15.0)	15.6 (15.2 to 15.9)	16.8 (16.4 to 17.1)	16.2 (15.8 to 16.6)	18.9 (18.5 to 19.3)	19.3 (18.8 to 19.7)	20.2 (19.7 to 20.6)	20.4 (19.9 to 20.8)	**0.761 (0.678 to 0.845)**	**0.516 (0.329 to 0.704)**	**-0.245 (-0.450 to -0.040)**
Subjective health level weighted % (95% CI)												
High	27.0 (26.7 to 27.3)	26.2 (25.9 to 26.5)	27.0 (26.6 to 27.3)	27.6 (27.3 to 27.9)	25.1 (24.8 to 25.4)	29.1 (28.8 to 29.5)	28.8 (28.5 to 29.1)	30.4 (30.0 to 30.7)	31.7 (31.4 to 32.0)	**0.217 (0.139 to 0.296)**	**0.971 (0.817 to 1.126)**	**0.754 (0.580 to 0.927)**
Normal	22.4 (22.1 to 22.7)	21.7 (21.4 to 22.0)	22.4 (22.2 to 22.7)	22.6 (22.3 to 22.9)	21.7 (21.4 to 22.0)	24.4 (24.1 to 24.7)	22.6 (22.3 to 22.9)	25.1 (24.7 to 25.4)	25.8 (25.5 to 26.2)	**0.278 (0.211 to 0.346)**	**0.659 (0.522 to 0.795)**	**0.380 (0.228 to 0.532)**
Low	10.0 (9.7 to 10.3)	10.2 (9.9 to 10.5)	10.5 (10.2 to 10.8)	10.5 (10.2 to 10.8)	12.1 (11.7 to 12.4)	12.9 (12.5 to 13.2)	11.4 (11.0 to 11.8)	14.1 (13.7 to 14.5)	13.1 (12.7 to 13.4)	**0.559 (0.484 to 0.634)**	**0.275 (0.119 to 0.431)**	**-0.284 (-0.457 to -0.111)**

*BMI* body mass index, *CI* confidence interval, *KCHS* Korea Community Health Survey.

The beta values were multiplied by 100 as a result of their minimal number. Numbers in bold indicate a significant difference (P < 0.05).

*The model was adjusted for age (19 to 39, 40 to 49, 50 to 59, 60 to 69, and ≥ 70 years), sex, body mass index (BMI; underweight, normal weight, overweight, and obese), residential areas (urban and rural), household income (lowest quartile, second quartile, third quartile, and highest quartile), educational level (elementary school or less, middle school, high school, and college or more), smoking status (non-smoker, ex-smoker, and current smoker), alcohol consumption (below a day, once to four days, and five days or more per month), and subjective health level (high, normal, and low).

**Table 4:** Prevalence of food labeling usage in the KCHS, 2014–2022 (n = 1,756,847).

Characteristic	2014	2015	2016	2017	2018	2019	2020	2021	2022	before the pandemic, β (2014–2019)	after the pandemic, β (2019–2022)	Trend difference, βdiff
Overall weighted % (95% CI)	17.6 (17.4 to 17.7)	17.0 (16.9 to 17.2)	17.6 (17.4 to 17.7)	17.7 (17.5 to 17.9)	16.9 (16.7 to 17.1)	18.8 (18.7 to 19.0)	19.7 (19.6 to 19.9)	21.0 (20.8 to 21.2)	21.3 (21.1 to 21.5)	**0.174 (0.133 to 0.215)**	**0.867 (0.784 to 0.949)**	**0.693 (0.601 to 0.785)**
Age group, year weighted % (95% CI)												
19–39	28.9 (28.6 to 29.3)	27.5 (27.1 to 27.9)	28.6 (28.2 to 29.0)	28.7 (28.3 to 29.1)	27.4 (26.9 to 27.8)	30.1 (29.7 to 30.6)	31.0 (30.6 to 31.5)	31.4 (30.9 to 31.9)	32.7 (32.3 to 33.2)	**0.124 (0.027 to 0.221)**	**0.817 (0.609 to 1.025)**	**0.693 (0.463 to 0.923)**
40–49	24.7 (24.3 to 25.1)	24.8 (24.4 to 25.2)	25.4 (24.9 to 25.8)	27.1 (26.7 to 27.5)	24.9 (24.4 to 25.4)	28.9 (28.3 to 29.4)	29.5 (28.9 to 30.0)	30.9 (30.4 to 31.5)	31.1 (30.5 to 31.6)	**0.639 (0.528 to 0.750)**	**0.803 (0.557 to 1.048)**	**0.164 (-0.106 to 0.433)**
50–59	15.3 (15.0 to 15.7)	15.7 (15.3 to 16.0)	16.7 (16.4 to 17.0)	17.5 (17.2 to 17.9)	17.2 (16.8 to 17.6)	21.3 (20.9 to 21.8)	22.4 (21.9 to 22.8)	24.6 (24.1 to 25.0)	25.1 (24.7 to 25.6)	**0.984 (0.894 to 1.075)**	**1.362 (1.161 to 1.564)**	**0.378 (0.157 to 0.599)**
60–69	7.2 (7.0 to 7.5)	8.4 (8.1 to 8.7)	9.0 (8.7 to 9.3)	9.7 (9.4 to 10.0)	10.6 (10.3 to 11.0)	13.6 (13.3 to 14.0)	14.5 (14.1 to 14.8)	17.0 (16.6 to 17.3)	18.0 (17.6 to 18.4)	**1.131 (1.056 to 1.207)**	**1.552 (1.388 to 1.716)**	**0.421 (0.240 to 0.602)**
≥ 70	2.4 (2.3 to 2.6)	2.8 (2.7 to 3.0)	2.9 (2.7 to 3.0)	3.0 (2.8 to 3.2)	3.5 (3.3 to 3.7)	5.1 (4.9 to 5.3)	5.0 (4.8 to 5.2)	6.1 (5.9 to 6.4)	6.6 (6.4 to 6.9)	**0.455 (0.411 to 0.500)**	**0.579 (0.482 to 0.675)**	**0.123 (0.017 to 0.230)**
Sex weighted % (95% CI)												
Male	9.3 (9.2 to 9.5)	9.2 (9.0 to 9.3)	9.7 (9.5 to 9.9)	9.8 (9.6 to 10.0)	9.2 (9.0 to 9.4)	11.5 (11.2 to 11.7)	12.9 (12.6 to 13.1)	14.0 (13.7 to 14.2)	14.4 (14.2 to 14.7)	**0.298 (0.251 to 0.345)**	**1.006 (0.903 to 1.109)**	**0.708 (0.595 to 0.822)**
Female	24.8 (24.5 to 25.0)	23.9 (23.6 to 24.1)	24.4 (24.2 to 24.7)	24.5 (24.2 to 24.7)	23.4 (23.1 to 23.7)	24.8 (24.5 to 25.0)	25.4 (25.1 to 25.7)	26.8 (26.5 to 27.1)	27.0 (26.7 to 27.3)	-0.035 (-0.097 to 0.027)	**0.815 (0.693 to 0.936)**	**0.850 (0.713 to 0.986)**
BMI group weighted % (95% CI)												
Underweight	21.6 (20.9 to 22.4)	20.0 (19.3 to 20.8)	19.8 (19.0 to 20.5)	20.3 (19.5 to 21.1)	19.6 (18.7 to 20.6)	22.2 (21.3 to 23.2)	20.8 (19.9 to 21.8)	22.5 (21.6 to 23.4)	22.4 (21.5 to 23.3)	0.022 (-0.179 to 0.222)	0.227 (-0.181 to 0.634)	0.205 (-0.249 to 0.659)
Normal weight	20.2 (19.9 to 20.4)	19.5 (19.3 to 19.8)	20.1 (19.9 to 20.4)	20.4 (20.1 to 20.7)	19.5 (19.2 to 19.8)	22.0 (21.6 to 22.3)	22.1 (21.8 to 22.4)	23.0 (22.7 to 23.3)	23.4 (23.1 to 23.7)	**0.241 (0.174 to 0.308)**	**0.535 (0.397 to 0.673)**	**0.294 (0.141 to 0.447)**
Overweight	15.4 (15.0 to 15.7)	14.8 (14.5 to 15.1)	15.5 (15.2 to 15.8)	15.5 (15.2 to 15.8)	15.0 (14.6 to 15.3)	16.9 (16.6 to 17.3)	18.0 (17.6 to 18.3)	19.4 (19.0 to 19.8)	19.6 (19.2 to 20.0)	**0.233 (0.155 to 0.312)**	**0.937 (0.775 to 1.099)**	**0.704 (0.524 to 0.883)**
Obese	14.1 (13.8 to 14.4)	14.3 (14.0 to 14.6)	15.0 (14.7 to 15.3)	15.1 (14.8 to 15.4)	14.7 (14.4 to 15.0)	16.4 (16.2 to 16.7)	17.8 (17.5 to 18.2)	19.4 (19.1 to 19.7)	19.7 (19.4 to 20.0)	**0.379 (0.309 to 0.449)**	**1.140 (1.000 to 1.280)**	**0.760 (0.604 to 0.917)**
Residential areas weighted % (95% CI)												
Urban	20.9 (20.7 to 21.1)	20.4 (20.2 to 20.6)	21.1 (20.9 to 21.3)	21.5 (21.2 to 21.7)	19.9 (19.6 to 20.1)	22.4 (22.1 to 22.6)	23.2 (22.9 to 23.4)	24.3 (24.1 to 24.6)	24.5 (24.3 to 24.8)	**0.167 (0.111 to 0.224)**	**0.759 (0.646 to 0.872)**	**0.592 (0.466 to 0.718)**
Rural	12.8 (12.6 to 13.0)	12.3 (12.1 to 12.6)	12.8 (12.5 to 13.0)	12.6 (12.4 to 12.8)	12.0 (11.8 to 12.3)	13.7 (13.5 to 14.0)	14.7 (14.4 to 14.9)	16.0 (15.7 to 16.3)	16.4 (16.1 to 16.7)	**0.100 (0.044 to 0.155)**	**0.928 (0.811 to 1.044)**	**0.828 (0.699 to 0.957)**
Household income weighted % (95% CI)												
Lowest quartile	6.2 (6.0 to 6.4)	6.2 (6.0 to 6.4)	6.2 (5.9 to 6.4)	6.1 (5.9 to 6.4)	5.5 (5.2 to 5.8)	6.7 (6.4 to 6.9)	7.7 (7.4 to 8.0)	8.8 (8.5 to 9.2)	7.8 (7.5 to 8.2)	0.001 (-0.060 to 0.063)	**0.477 (0.338 to 0.616)**	**0.476 (0.324 to 0.627)**
Second quartile	16.0 (15.8 to 16.3)	15.2 (14.9 to 15.4)	15.6 (15.4 to 15.9)	14.9 (14.6 to 15.2)	12.9 (12.6 to 13.2)	15.2 (14.9 to 15.5)	16.2 (15.9 to 16.5)	17.2 (16.9 to 17.5)	17.1 (16.8 to 17.4)	**-0.340 (-0.406 to -0.275)**	**0.681 (0.544 to 0.818)**	**1.021 (0.869 to 1.173)**
Third quartile	22.8 (22.4 to 23.1)	21.9 (21.5 to 22.2)	22.3 (21.9 to 22.6)	22.3 (21.9 to 22.6)	20.5 (20.1 to 20.8)	22.4 (22.0 to 22.8)	23.5 (23.1 to 23.9)	24.9 (24.5 to 25.3)	24.9 (24.5 to 25.3)	**-0.175 (-0.260 to -0.090)**	**0.886 (0.709 to 1.062)**	**1.060 (0.864 to 1.256)**
Highest quartile	26.1 (25.7 to 26.6)	25.9 (25.5 to 26.4)	25.9 (25.4 to 26.3)	27.0 (26.5 to 27.4)	23.7 (23.3 to 24.1)	26.5 (26.1 to 26.9)	27.6 (27.2 to 28.0)	28.3 (27.9 to 28.7)	28.7 (28.3 to 29.0)	**-0.106 (-0.207 to -0.004)**	**0.709 (0.540 to 0.879)**	**0.815 (0.618 to 1.012)**
Educational level weighted % (95% CI)												
Elementary school or less	2.8 (2.7 to 3.0)	3.0 (2.8 to 3.1)	3.0 (2.8 to 3.1)	3.1 (2.9 to 3.2)	3.3 (3.1 to 3.5)	4.6 (4.4 to 4.8)	4.2 (4.0 to 4.4)	5.0 (4.8 to 5.2)	4.8 (4.5 to 5.0)	**0.284 (0.243 to 0.325)**	**0.132 (0.040 to 0.225)**	**-0.151 (-0.252 to -0.050)**
Middle school	8.1 (7.8 to 8.5)	8.4 (8.0 to 8.7)	9.0 (8.7 to 9.4)	8.8 (8.4 to 9.1)	8.9 (8.5 to 9.3)	11.8 (11.3 to 12.2)	11.5 (11.1 to 11.9)	12.6 (12.2 to 13.1)	13.1 (12.7 to 13.6)	**0.546 (0.455 to 0.637)**	**0.523 (0.324 to 0.721)**	-0.023 (-0.242 to 0.195)
High school	17.8 (17.5 to 18.1)	17.2 (16.9 to 17.5)	17.6 (17.3 to 17.9)	17.8 (17.5 to 18.0)	16.7 (16.4 to 17.0)	19.8 (19.4 to 20.1)	20.1 (19.7 to 20.4)	21.4 (21.1 to 21.8)	21.7 (21.4 to 22.1)	**0.225 (0.149 to 0.300)**	**0.723 (0.569 to 0.877)**	**0.498 (0.327 to 0.670)**
College or more	29.2 (28.9 to 29.5)	28.1 (27.8 to 28.4)	28.7 (28.4 to 29.0)	29.0 (28.7 to 29.3)	27.0 (26.7 to 27.4)	30.2 (29.9 to 30.6)	31.3 (31.0 to 31.7)	31.9 (31.6 to 32.3)	32.5 (32.1 to 32.8)	0.048 (-0.032 to 0.128)	**0.737 (0.579 to 0.894)**	**0.689 (0.512 to 0.866)**
Smoking status weighted % (95% CI)												
Non-smoker	22.8 (22.6 to 23.1)	22.1 (21.9 to 22.3)	22.7 (22.4 to 22.9)	22.6 (22.3 to 22.8)	21.5 (21.2 to 21.7)	23.3 (23.0 to 23.5)	23.5 (23.3 to 23.8)	25.1 (24.9 to 25.4)	25.6 (25.3 to 25.9)	0.010 (-0.046 to 0.066)	**0.853 (0.741 to 0.964)**	**0.843 (0.718 to 0.968)**
Ex-smoker	8.5 (8.2 to 8.8)	8.5 (8.2 to 8.8)	8.9 (8.7 to 9.2)	9.1 (8.9 to 9.4)	8.6 (8.3 to 8.9)	11.2 (10.9 to 11.6)	12.5 (12.1 to 12.9)	13.4 (13.1 to 13.8)	14.7 (14.3 to 15.0)	**0.412 (0.340 to 0.484)**	**1.119 (0.967 to 1.272)**	**0.708 (0.539 to 0.876)**
Current smoker	9.1 (8.9 to 9.4)	8.7 (8.4 to 8.9)	9.5 (9.2 to 9.8)	9.5 (9.2 to 9.8)	9.2 (8.9 to 9.5)	11.1 (10.7 to 11.4)	12.5 (12.1 to 12.9)	13.3 (12.9 to 13.7)	13.9 (13.5 to 14.3)	**0.303 (0.230 to 0.376)**	**0.931 (0.763 to 1.099)**	**0.628 (0.445 to 0.812)**
Alcohol consumption, days/month weighted % (95% CI)												
< 1	17.8 (17.6 to 18.1)	17.2 (17.0 to 17.5)	17.5 (17.3 to 17.7)	17.0 (16.8 to 17.2)	16.7 (16.4 to 16.9)	18.0 (17.8 to 18.3)	19.1 (18.8 to 19.3)	20.7 (20.4 to 20.9)	20.5 (20.3 to 20.8)	-0.031 (-0.090 to 0.027)	**0.917 (0.805 to 1.029)**	**0.948 (0.822 to 1.075)**
1–4	21.8 (21.5 to 22.1)	21.0 (20.7 to 21.3)	21.7 (21.4 to 22.0)	22.3 (22.0 to 22.6)	20.6 (20.3 to 21.0)	23.4 (23.0 to 23.7)	24.0 (23.7 to 24.4)	24.9 (24.5 to 25.3)	26.1 (25.7 to 26.4)	**0.205 (0.125 to 0.285)**	**0.886 (0.719 to 1.054)**	**0.681 (0.496 to 0.867)**
≥ 5	11.2 (10.9 to 11.5)	11.2 (10.9 to 11.5)	12.0 (11.7 to 12.3)	13.0 (12.7 to 13.2)	12.4 (12.1 to 12.7)	14.5 (14.2 to 14.9)	15.2 (14.8 to 15.6)	16.3 (15.9 to 16.7)	16.3 (15.9 to 16.7)	**0.593 (0.518 to 0.668)**	**0.624 (0.454 to 0.794)**	0.031 (-0.155 to 0.217)
Subjective health level weighted % (95% CI)												
High	20.7 (20.4 to 20.9)	21.3 (21.0 to 21.6)	21.9 (21.7 to 22.2)	22.5 (22.2 to 22.7)	20.5 (20.2 to 20.8)	23.6 (23.3 to 24.0)	23.9 (23.6 to 24.2)	25.6 (25.2 to 25.9)	26.7 (26.4 to 27.1)	**0.154 (0.080 to 0.227)**	**1.118 (0.972 to 1.264)**	**0.964 (0.801 to 1.128)**
Normal	24.9 (24.5 to 25.3)	17.4 (17.1 to 17.6)	18.0 (17.8 to 18.3)	18.1 (17.8 to 18.3)	17.1 (16.9 to 17.4)	19.4 (19.2 to 19.7)	18.4 (18.1 to 18.6)	20.7 (20.4 to 20.9)	21.1 (20.8 to 21.4)	**0.178 (0.116 to 0.240)**	**0.715 (0.589 to 0.842)**	**0.537 (0.397 to 0.678)**
Low	16.3 (15.9 to 16.7)	8.0 (7.7 to 8.2)	8.3 (8.0 to 8.6)	8.1 (7.9 to 8.4)	9.5 (9.2 to 9.8)	10.0 (9.7 to 10.3)	9.0 (8.7 to 9.4)	11.3 (11.0 to 11.7)	10.4 (10.1 to 10.8)	**0.435 (0.368 to 0.502)**	**0.298 (0.158 to 0.439)**	-0.136 (-0.292 to 0.019)

*BMI* body mass index, *CI* confidence interval, *KCHS* Korea Community Health Survey.

The beta values were multiplied by 100 as a result of their minimal number. Numbers in bold indicate a significant difference (P < 0.05).

*The model was adjusted for age (19 to 39, 40 to 49, 50 to 59, 60 to 69, and ≥ 70 years), sex, body mass index (BMI; underweight, normal weight, overweight, and obese), residential areas (urban and rural), household income (lowest quartile, second quartile, third quartile, and highest quartile), educational level (elementary school or less, middle school, high school, and college or more), smoking status (non-smoker, ex-smoker, and current smoker), alcohol consumption (below a day, once to four days, and five days or more per month), and subjective health level (high, normal, and low).

The upward slope in overall food labeling awareness became less pronounced and even exhibited a downward slope momentarily during the pandemic. The slope value was 2.158 (95% CI 2.105–2.210) before the pandemic and 0.399 (95% CI, 0.297 to 0.501) during the pandemic (β_diff_ − 1.759; 95% CI − 1.874 to − 1.644). (Table 2) Before the pandemic, the rate of food labeling comprehension exhibited a gradual increase, which intensifies during the pandemic. The overall slope value was 0.259 (95% CI 0.215–0.303) before the pandemic and 0.794 (95% CI 0.705–0.882) during the pandemic (β_diff_ 0.535; 95% CI 0.436–0.634). (Table 3) Similarly, regarding food labeling usage, the overall slope was 0.174 (95% CI 0.133–0.215) before the pandemic and 0.867 (95% CI 0.784–0.949) during the pandemic (β_diff_ 0.693; 95% CI 0.601–0.785), showing a similar trend change. (Table 4) Adjusted OR of food labeling awareness was 0.900 (95% CI 0.887–0.914) between 2019 and 2020, 1.013 (95% CI 0.998–1.028) between 2020 and 2021, and 1.111 (1.095–1.128) between 2021 and 2022 (Table S5). Conversely adjusted OR of food labeling comprehension was 0.964 (95% CI 0.948–0.980) between 2019 and 2020, 1.082 (95% CI 1.064–1.100) between 2020 and 2021, and 1.050 (95% CI 1.033–1.067) between 2021 and 2022 (Table S6). Similarly adjusted OR of food labeling usage was 0.995 (95% CI 0.977–1.013) between 2019 and 2020, 1.091 (95% CI 1.072–1.110) between 2020 and 2021, and 1.041 (95% CI 1.023–1.059) between 2021 and 2022 (Table S7).

The ratio of OR before to during the pandemic increased in relation to the age group (≥ 65 versus 19–39 years; ratio of OR 1.295; 95% CI 1.270–1.319), being male (ratio of OR 1.179; 95% CI 1.164–1.194), higher BMI (obese versus underweight; ratio of OR 1.237; 95% CI 1.198–1.278), residing in a rural area (ratio of OR 1.062; 95% CI 1.049–1.076); having a lower household income (ratio of OR 1.091; 95% CI, 1.067–1.117), lower educational level (risk of OR 1.163; 95% CI 1.138–1.189), being a smoker (ratio of OR 1.199; 95% CI 1.178–1.220), having heavy alcohol consumption (risk of OR 1.103; 95% CI 1.085–1.123) (Table 5).

**Table 5:** Pandemic-related factors of food labeling awareness in the KCHS, 2014–2022 (n = 1,756,847).

	Adjusted OR (95% CI)*			Ratio of OR (95% CI)∥
	Overall (n = 1,756,847)	pre-COVID pandemic (n = 1,217,443)	COVID-19 pandemic (n = 539,404)	
Age group, year				
19–39	1.000 (reference)	1.000 (reference)	1.000 (reference)	1.000 (reference)
40–64	0.593 (0.588 to 0.597)	0.566 (0.561 to 0.571)	0.651 (0.641 to 0.661)	**1.150 (1.130 to 1.171)**
≥ 65	0.149 (0.147 to 0.150)	0.129 (0.128 to 0.131)	0.167 (0.165 to 0.170)	**1.295 (1.270 to 1.319)**
Sex				
Male	0.611 (0.607 to 0.614)	0.581 (0.577 to 0.585)	0.685 (0.677 to 0.692)	**1.179 (1.164 to 1.194)**
Female	1.000 (reference)	1.000 (reference)	1.000 (reference)	1.000 (reference)
BMI group				
Underweight	1.000 (reference)	1.000 (reference)	1.000 (reference)	1.000 (reference)
Normal weight	1.147 (1.130 to 1.163)	1.117 (1.098 to 1.137)	1.214 (1.182 to 1.247)	**1.087 (1.053 to 1.122)**
Overweight	0.998 (0.983 to 1.013)	0.944 (0.927 to 0.961)	1.119 (1.089 to 1.150)	**1.185 (1.147 to 1.225)**
Obese	1.041 (1.026 to 1.057)	0.969 (0.952 to 0.986)	1.199 (1.166 to 1.232)	**1.237 (1.198 to 1.278)**
Residential areas				
Urban	1.000 (reference)	1.000 (reference)	1.000 (reference)	1.000 (reference)
Rural	0.571 (0.567 to 0.574)	0.561 (0.557 to 0.565)	0.596 (0.590 to 0.603)	**1.062 (1.049 to 1.076)**
Household income				
Lowest quartile	0.164 (0.163 to 0.166)	0.164 (0.162 to 0.166)	0.179 (0.175 to 0.182)	**1.091 (1.067 to 1.117)**
Second quartile	0.453 (0.450 to 0.457)	0.467 (0.462 to 0.472)	0.455 (0.448 to 0.461)	0.974 (0.957 to 0.992)
Third quartile	0.777 (0.770 to 0.784)	0.799 (0.790 to 0.807)	0.794 (0.781 to 0.806)	0.994 (0.975 to 1.013)
Highest quartile	1.000 (reference)	1.000 (reference)	1.000 (reference)	1.000 (reference)
Educational level				
Elementary school or less	0.110 (0.109 to 0.111)	0.104 (0.103 to 0.106)	0.121 (0.119 to 0.123)	**1.163 (1.138 to 1.189)**
Middle school	0.291 (0.288 to 0.294)	0.278 (0.275 to 0.282)	0.316 (0.310 to 0.322)	**1.137 (1.111 to 1.163)**
High school	0.570 (0.566 to 0.574)	0.560 (0.555 to 0.565)	0.591 (0.582 to 0.599)	**1.055 (1.038 to 1.073)**
College or more	1.000 (reference)	1.000 (reference)	1.000 (reference)	1.000 (reference)
Smoking status				
Non-smoker	1.000 (reference)	1.000 (reference)	1.000 (reference)	1.000 (reference)
Ex-smoker	0.564 (0.560 to 0.569)	0.520 (0.515 to 0.525)	0.660 (0.651 to 0.669)	**1.269 (1.248 to 1.291)**
Current smoker	0.674 (0.668 to 0.679)	0.644 (0.638 to 0.650)	0.772 (0.761 to 0.784)	**1.199 (1.178 to 1.220)**
Alcohol consumption, days/month				
< 1	1.000 (reference)	1.000 (reference)	1.000 (reference)	1.000 (reference)
1–4	1.710 (1.698 to 1.722)	1.732 (1.718 to 1.747)	1.784 (1.761 to 1.807)	**1.030 (1.014 to 1.046)**
≥ 5	1.025 (1.017 to 1.033)	1.025 (1.015 to 1.034)	1.131 (1.115 to 1.148)	**1.103 (1.085 to 1.123)**
Subjective health level				
High	1.333 (1.324 to 1.342)	1.309 (1.299 to 1.320)	1.341 (1.325 to 1.358)	**1.024 (1.010 to 1.040)**
Normal	1.000 (reference)	1.000 (reference)	1.000 (reference)	1.000 (reference)
Low	0.401 (0.397 to 0.404)	0.396 (0.392 to 0.400)	0.416 (0.410 to 0.423)	**1.051 (1.031 to 1.070)**

*CI* confidence interval, *BMI* body mass index, *KCHS* Korea Community Health Survey, *OR* odds ratio.

Numbers in bold indicate a significant difference (P < 0.05).

*The model was adjusted for age (19 to 39, 40 to 49, 50 to 59, 60 to 69, and ≥ 70 years), sex, body mass index (BMI; underweight, normal weight, overweight, and obese), residential areas (urban and rural), household income (lowest quartile, second quartile, third quartile, and highest quartile), educational level (elementary school or less, middle school, high school, and college or more), smoking status (non-smoker, ex-smoker, and current smoker), alcohol consumption (below a day, once to four days, and five days or more per month), and subjective health level (high, normal, and low).

∥The COVID-19 pandemic period versus the pre-pandemic period.

## Discussion

### Findings of our study

This study analyzed the 9-year trend in the prevalence of food labeling awareness, food labeling comprehension, and food labeling usage based on nationally representative data from adults in South Korea from 2014 to 2022. The overall prevalence of food labeling awareness, food labeling comprehension, and food labeling usage exhibited a continuously increasing trend. Unlike the trend of before the pandemic breakout, there was a deceleration in the trend slope of nutrition label awareness, indicating a slower rate of increase during the pandemic period from 2019 to 2022. On the other hand, the trend slope of food labeling comprehension and usage accelerated, indicating a faster rate of increase during the same period of pandemic. The pandemic-related vulnerability factors of food labeling awareness were older age, male, obesity, residing in rural area, lower household income, lower educational level, smoking, and increased alcohol consumption. This study’s findings suggest personalized nutrition strategies, such as educating the importance of a balanced diet to recognize vulnerable groups with risk factors, and to improve food labeling awareness among Korean adults after the pandemic breakout.

### Comparison with previous studies

Few studies have investigated food labeling awareness or the determination of vulnerability factors. A previous Korean study on the prevalence of food labeling reported a constant increase in awareness and utilization of food labeling from 2014 to 2017^18^. However, this study only analyzed a short period (2014–2017) that did not include the pandemic period. In addition, studies conducted in China investigating the current food labeling knowledge, attitude, and practice^27^ only conducted a survey in a single city with a small sample size (n = 636) and cross-sectional design, and were unable to demonstrate any trend changes. A study conducted in the USA analyzed the food labeling usage of participants with chronic diseases^28^. However, this study exclusively focused on participants with chronic diseases, limiting its ability to provide insights into food labeling usage among the general population. In addition, a study from Italy discovered that most people do not use food labeling completely consciously and that simplifying the label format would help people with no expertise to read the labeling^8^. However, the small sample size and short observation period has a difficulty showing the trend of the general population. Results from the present study provide long-term evidence demonstrating how the pandemic affected the awareness, comprehension, and usage of food labeling, which covered 1,756,847 participants and was collected over nine years.

### Possible explanations

The constant increase of food labeling awareness, comprehension, and usage despite the unchanging food labeling system may be explained by increasing interest of people towards maintaining a healthy diet. The deceleration of awareness increases and the acceleration of the increase in food labeling comprehension and usage may be attributable to quarantine and social distancing^29^. A study conducted in Korea revealed that there was a substantial rise in the volume of business for food services (839.7%), food and beverage (203.8%), as well as agricultural goods, meat, and fish products (193.5%) from 2017 to 2021^30^. As a result of the pandemic, a greater number of individuals have resorted to shopping for groceries through the internet (also referred to as online shopping). This movement might have likely hindered the increase in food labeling awareness, as fewer individuals see the product in person and inspect the nutrition label^31,32^. Additionally, after selecting the product and delivering it, examining the package becomes irrelevant, thereby reducing the increase in awareness. However, individuals who were already aware of food labeling might have become more active in reading and utilizing food labeling, as the pandemic has heightened people’s interest in maintaining a healthy diet^33^. Furthermore, because of the COVID-19 quarantine, a greater number of individuals have started cooking meals at home as opposed to dining out^32,34^. This shift in behavior may have led to an increase in comprehension and usage of food labeling.

### Policy implication

As previous studies have not analyzed the differences in food labeling awareness before and during the pandemic, they exhibit a somewhat optimistic view of the trend in food labeling awareness^18,33^. However, this study’s results imply that the pandemic has negatively influenced the overall awareness of food labeling, necessitating further efforts to increase its awareness^21^. There is no specific advertisement that would assist in increasing the awareness of food labeling. Food labeling awareness significantly affects whether a consumer can maintain a healthy diet when required. Another suggested policy is the front-of-pack labeling (FOPL)^35^. It indicates that people with lower socioeconomic status (defined according to participants’ education, income, areas of residence or the store’s location) are affected more by FOPL. Policymakers should recognize that the pandemic negatively affected people’s food labeling awareness and that efforts must be made to provide citizens with the right information on the label of food packages^36–38^.

### Strength and limitations

This study included a large-scale, nationally representative, and long-term serial survey that covered the pandemic period (2019–2022). However, there were limitations to this study that should be considered. First, the data utilized by the researchers were missing for people who did not answer all the questions related to the covariates used in this study. The missing data may lead to a biased interpretation of the data as the missing data may have similar factors^4,39^. Second, while this study contained data from the pre-pandemic (2014–2019) to the pandemic (2020–2022) period, it did not contain data from the post pandemic (2023–) period^40^. Further research of food labeling awareness, comprehension, and usage must be conducted to estimate the future trend. Third, this research examines the trend of food labeling awareness, comprehension, and usage before and after the pandemic, but does not examine the causal relationship between the two factors. Therefore, further research must be conducted to examine the causal relationship and solve the fundamental problem of information inequality in food labeling. Finally, the dataset exclusively comprised individuals in Korea, thereby missing the consideration of racial and cultural variances in the research^41^. Consequently, additional investigations should be conducted in countries with diverse cultural backgrounds.

## Conclusion

This study elucidated that food labeling awareness, comprehension, and usage increased throughout the years of data collection. The COVID-19 pandemic negatively influenced food labeling awareness but positively impacted food labeling comprehension and usage. Through the identification of risk factors for food labeling awareness, it will be necessary to present a political solution to steadily increase food labeling awareness and bridge the gap between the groups.

### Supplementary Information

Supplementary Information.

## Data Availability

The data are available upon request. Study protocol and statistical code: Available from DKY (yonkkang@gmail.com). Data set: Available from the Korean Disease Control and Prevention Agency (KDCA) through a data use agreement.
