# Supplementary material for: National prevalence and trends in food labeling awareness, comprehension, usage, and COVID-19 pandemic-related factors in South Korea, 2014–2022
Source: Sci Rep. 2024 Jan 31;14:2617. doi: 10.1038/s41598-024-51948-1 (PMC10831073; doi:10.1038/s41598-024-51948-1)
Supplement: Supplementary file 1 — Supplementary Information. [file 41598_2024_51948_MOESM1_ESM.docx]

| **Supplementary Material** |
| --- |

Original paper

**National prevalence and trends in food labeling awareness, comprehension, usage, and COVID-19 pandemic-related factors in South Korea, 2014–2022**

Running title: **Food labeling awareness**

Yujin Choi,^1,2∥^ Hyeon Jin Kim,^1,3*^ Jaeyu Park,^1,3∥^ Seung Won Lee,^4^ Masoud Rahmati,^5,6^ Ai Koyanagi,^7^ Lee Smith,^8^ Min Seo Kim,^9^ [Guillermo F López Sánchez](https://streaklinks.com/BfMTWjzVFXnjyxjmNQoEXiV-/https%3A%2F%2Fpubmed.ncbi.nlm.nih.gov%2F%3Fsort%3Ddate%26size%3D200%26term%3DL%25C3%25B3pez%2BS%25C3%25A1nchez%2BGF%26cauthor_id%3D36808652?email=yonkkang%40gmail.com),^10^ Dragioti Elena,^11,12^ Jinseok Lee,^13^ Sang Youl Rhee,^1,14^ Sunyoung Kim,^15^ Hyunjung Lim,^16*^ Dong Keon Yon,^1,3,17^*

∥ These authors contributed equally

***Corresponding authors**

Contents of supplementary appendix

| Supplementary material | Supplementary methodology | p.3 |
| --- | --- | --- |
| Table S1 | Baseline in the KCHS, 2014–2022 (n =1,756,847). | p.4-7 |
| Table S2 | Prevalence of food labeling awareness in the KCHS with SE, 2014–2022 (n=1,756,847). | p.8-11 |
| Table S3 | Prevalence of food labeling comprehension in the KCHS with SE, 2014–2022 (n =1,756,847). | p.12-15 |
| Table S4 | Prevalence of food labeling usage in the KCHS with SE, 2014–2022 (n =1,756,847). | p.16-19 |
| Table S5 | Adjusted odds ratios of food labeling awareness among pre-pandemic and during the pandemic, ORs (95% CI), in the data gathered from the KCHS. | p.20-22 |
| Table S6 | Adjusted odds ratios of food labeling comprehension among pre-pandemic and during the pandemic, ORs (95% CI), in the data gathered from the KCHS. | p.23-25 |
| Table S7 | Adjusted odds ratios of food labeling usage among pre-pandemic and during the pandemic, ORs (95% CI), in the data gathered from the KCHS. | p.26-28 |

**Supplementary methodology**

The methodological estimating equations of three linear probability models are written down below.

[Food labeling awareness]

Crude model:

awareness=β0+β1×year+ϵ

Adjusted model:

awareness= β0+β1×year+β2×age+β3×sex+β4×bmi+β5×residential area+β6×household income+β7×educational level+β8×smoking status+β9×alcohol consumption+β10×subjective health level+ϵ

[Food labeling comprehension]

Crude model:

comprehension=β0+β1×year+ϵ

Adjusted model:

comprehension = β0+β1×year+β2×age+β3×sex+β4×bmi+β5×residential area+β6×household income+β7×educational level+β8×smoking status+β9×alcohol consumption+β10×subjective health level+ϵ

[Food labeling usage]

Crude model:

usage =β0+β1×year+ϵ

Adjusted model:

usage = β0+β1×year+β2×age+β3×sex+β4×bmi+β5×residential area+β6×household income+β7×educational level+β8×smoking status+β9×alcohol consumption+β10×subjective health level+ϵ

**Table S1.** Baseline in the KCHS, 2014–2022 (n =1,756,847).

| **Characteristic** | **Food labeling Awareness** | | **Food labeling comprehension** | | **Food labeling usage** | |
| --- | --- | --- | --- | --- | --- | --- |
|  | **Not aware** | **Aware** | **Do not comprehend** | **Comprehend** | **Do not use** | **Use** |
| Overall | 859,645 (48.9) | 897,202 (51.1) | 1,355,721 (77.2) | 401,126 (22.8) | 1,431,105 (81.5) | 325,742 (18.5) |
| Age, year (mean, SD) | 59.7 (17.2) | 47.4 (15.4) | 55.9 (17.4) | 45.2 (14.5) | 55.4 (17.5) | 45.0 (14.2) |
| Age group, year (n, %) |  |  |  |  |  |  |
| 19-39 | 127,189 (14.8) | 292,471 (32.6) | 269,329 (19.9) | 150,331 (37.5) | 296,327 (20.7) | 123,333 (37.9) |
| 40-49 | 101,452 (11.8) | 197,529 (22.0) | 201,617 (14.9) | 97,364 (24.3) | 218,099 (15.2) | 80,882 (24.8) |
| 50-59 | 152,690 (17.8) | 193,665 (21.6) | 264,606 (19.5) | 81,749 (20.4) | 280,153 (19.6) | 66,202 (20.3) |
| 60-69 | 188,444 (21.9) | 140,461 (15.7) | 278,005 (20.5) | 50,900 (12.7) | 289,081 (20.2) | 39,824 (12.2) |
| ≥70 | 289,870 (33.7) | 73,076 (8.1) | 342,164 (25.2) | 20,782 (5.2) | 347,445 (24.3) | 15,501 (4.8) |
| Sex (n, %) |  |  |  |  |  |  |
| Male | 447,278 (52.0) | 357,530 (39.9) | 689,480 (50.9) | 115,328 (28.8) | 716,591 (50.1) | 88,217 (27.1) |
| Female | 412,367 (48.0) | 539,672 (60.2) | 666,241 (49.1) | 285,798 (71.3) | 714,514 (49.9) | 237,525 (72.9) |
| BMI group (n, %) |  |  |  |  |  |  |
| Underweight | 41,679 (4.9) | 40,622 (4.5) | 61,453 (4.5) | 20,848 (5.2) | 65,029 (4.5) | 17,272 (5.3) |
| Normal weight | 347,427 (40.4) | 388,347 (43.3) | 548,991 (40.5) | 186,783 (46.6) | 581,191 (40.6) | 154,583 (47.5) |
| Overweight | 218,705 (25.4) | 212,663 (23.7) | 342,656 (25.3) | 88,712 (22.1) | 359,952 (25.2) | 71,416 (21.9) |
| Obese | 251,834 (29.3) | 255,570 (28.5) | 402,621 (29.7) | 104,783 (26.1) | 424,933 (29.7) | 82,471 (25.3) |
| Residential areas (n, %) |  |  |  |  |  |  |
| Urban | 449,212 (52.3) | 589,748 (65.7) | 761,120 (56.1) | 277,840 (69.3) | 810,901 (56.7) | 228,059 (70.0) |
| Rural | 410,433 (47.7) | 307,454 (34.3) | 594,601 (43.9) | 123,286 (30.7) | 620,204 (43.3) | 97,683 (30.0) |
| Household income (n, %) |  |  |  |  |  |  |
| Lowest quartile | 227,873 (26.5) | 73,242 (8.2) | 274,537 (20.3) | 26,578 (6.6) | 280,943 (19.6) | 20,172 (6.2) |
| Second quartile | 308,985 (35.9) | 273,714 (30.5) | 467,811 (34.5) | 114,888 (28.6) | 491,911 (34.4) | 90,788 (27.9) |
| Third quartile | 185,136 (21.5) | 281,212 (31.3) | 336,366 (24.8) | 129,982 (32.4) | 360,362 (25.2) | 105,986 (32.5) |
| Highest quartile | 137,651 (16.0) | 269,034 (30.0) | 277,007 (20.4) | 129,678 (32.3) | 297,889 (20.8) | 108,796 (33.4) |
| Educational level (n, %) |  |  |  |  |  |  |
| Elementary school or less | 306,547 (35.7) | 75,704 (8.4) | 362,548 (26.7) | 19,703 (4.9) | 368,188 (25.7) | 14,063 (4.3) |
| Middle school | 123,377 (14.4) | 80,212 (8.9) | 175,975 (13.0) | 27,614 (6.9) | 183,009 (12.8) | 20,580 (6.3) |
| High school | 228,528 (26.6) | 291,291 (32.5) | 395,651 (29.2) | 124,168 (31.0) | 422,171 (29.5) | 97,648 (30.0) |
| College or more | 201,193 (23.4) | 449,995 (50.2) | 421,547 (31.1) | 229,641 (57.3) | 457,737 (32.0) | 193,451 (59.4) |
| Smoking status (n, %) |  |  |  |  |  |  |
| Non-smoker | 494,271 (57.5) | 616,721 (68.7) | 799,550 (59.0) | 311,442 (77.6) | 853,304 (59.6) | 257,688 (79.1) |
| Ex-smoker | 194,748 (22.7) | 137,078 (15.3) | 286,949 (21.2) | 44,877 (11.2) | 296,780 (20.7) | 35,046 (10.8) |
| Current smoker | 170,626 (19.9) | 143,403 (16.0) | 269,222 (19.9) | 44,807 (11.2) | 281,021 (19.6) | 33,008 (10.1) |
| Alcohol consumption, days/month (n, %) |  |  |  |  |  |  |
| <1 | 462,786 (53.8) | 411,037 (45.8) | 679,278 (50.1) | 194,545 (48.5) | 713,783 (49.9) | 160,040 (49.1) |
| 1-4 | 205,235 (23.9) | 311,659 (34.7) | 372,635 (27.5) | 144,259 (36.0) | 399,619 (27.9) | 117,275 (36.0) |
| ≥5 | 191,624 (22.3) | 174,506 (19.5) | 303,808 (22.4) | 62,322 (15.5) | 317,703 (22.2) | 48,427 (14.9) |
| Subjective health level (n, %) |  |  |  |  |  |  |
| High | 274,255 (31.9) | 403,473 (45.0) | 487,510 (36.0) | 190,218 (47.4) | 521,109 (36.4) | 156,619 (48.1) |
| Normal | 355,081 (41.3) | 391,869 (43.7) | 574,239 (42.4) | 172,711 (43.1) | 607,877 (42.5) | 139,073 (42.7) |
| Low | 230,309 (26.8) | 101,860 (11.4) | 293,972 (21.7) | 38,197 (9.5) | 302,119 (21.1) | 30,050 (9.2) |

BMI, body mass index; KCHS, Korea Community Health Survey; SD, standard deviation.

**Table S2.** Prevalence of food labeling awareness in the KCHS with SE, 2014–2022 (n=1,756,847).

|  | **2014** | **2015** | **2016** | **2017** | **2018** | **2019** | **2020** | **2021** | **2022** | **before the pandemic, β (2014-2019)** | **after the pandemic, β (2019-2022)** | **Trend difference, βdiff** |
| --- | --- | --- | --- | --- | --- | --- | --- | --- | --- | --- | --- | --- |
| Overall weighted % ±SE | 44.6±0.107 | 45.7±0.107 | 47.7±0.108 | 48.6±0.107 | 51.3±0.120 | 56.3±0.117 | 55.5±0.118 | 55.8±0.118 | 57.6±0.115 | **2.16±0.027** | **0.40±0.052** | **-1.76±2.290** |
| Age group, year weighted% ±SE |  |  |  |  |  |  |  |  |  |  |  |  |
| 19-39 | 64.3±0.198 | 64.4±0.203 | 66.9±0.200 | 68.9±0.201 | 70.4±0.223 | 75.6±0.219 | 74.2±0.219 | 73.4±0.225 | 75.8±0.221 | **2.12±0.050** | -0.03±0.099 | **-2.15±3.608** |
| 40-49 | 58.8±0.237 | 60.0±0.242 | 63.0±0.241 | 65.3±0.242 | 66.3±0.276 | 74.2±0.265 | 71.9±0.273 | 71.6±0.278 | 72.9±0.274 | **2.71±0.061** | **-0.42±0.121** | **-3.14±4.654** |
| 50-59 | 44.4±0.236 | 47.5±0.237 | 50.4±0.237 | 52.5±0.238 | 56.4±0.266 | 65.6±0.258 | 64.1±0.261 | 64.4±0.264 | 67.2±0.257 | **3.78±0.059** | **0.50±0.115** | **-3.28±4.402** |
| 60-69 | 27.1±0.242 | 32.0±0.244 | 33.7±0.245 | 36.3±0.242 | 42.3±0.272 | 51.7±0.265 | 50.7±0.265 | 52.7±0.258 | 56.1±0.247 | **4.48±0.061** | **1.55±0.115** | **-2.93±3.589** |
| ≥70 | 11.0±0.167 | 13.6±0.177 | 14.0±0.177 | 15.5±0.176 | 19.9±0.212 | 25.0±0.206 | 24.3±0.210 | 25.8±0.213 | 28.3±0.210 | **2.63±0.046** | **1.15±0.093** | **-1.49±5.952** |
| Sex weighted % ±SE |  |  |  |  |  |  |  |  |  |  |  |  |
| Male | 36.8±0.152 | 37.9±0.153 | 40.1±0.155 | 41.1±0.155 | 43.8±0.175 | 51.6±0.177 | 50.6±0.176 | 50.4±0.177 | 52.5±0.173 | **2.58±0.039** | **0.26±0.078** | **-2.32±5.838** |
| Female | 51.3±0.148 | 52.5±0.147 | 54.2±0.147 | 55.1±0.145 | 57.7±0.161 | 60.1±0.155 | 59.6±0.157 | 60.2±0.157 | 61.7±0.153 | **1.70±0.036** | **0.55±0.069** | **-1.15±4.832** |
| BMI group weighted % ±SE |  |  |  |  |  |  |  |  |  |  |  |  |
| Underweight | 46.0±0.466 | 45.9±0.476 | 46.3±0.482 | 47.2±0.486 | 50.9±0.593 | 55.9±0.567 | 51.3±0.576 | 51.7±0.560 | 53.7±0.544 | **1.75±0.124** | **-0.58±0.249** | **-2.33±4.311** |
| Normal weight | 47.1±0.160 | 48.3±0.162 | 49.8±0.164 | 50.9±0.164 | 53.8±0.189 | 58.9±0.191 | 56.6±0.183 | 56.5±0.184 | 58.2±0.180 | **2.11±0.042** | **-0.17±0.083** | **-2.27±2.972** |
| Overweight | 42.2±0.216 | 43.4±0.215 | 45.7±0.216 | 46.4±0.214 | 49.4±0.242 | 55.3±0.241 | 54.3±0.238 | 54.6±0.238 | 56.3±0.233 | **2.35±0.054** | **0.36±0.106** | **-1.99±3.187** |
| Obese | 41.9±0.213 | 43.6±0.210 | 46.2±0.206 | 47.4±0.205 | 49.6±0.213 | 54.4±0.199 | 55.7±0.214 | 56.3±0.216 | 58.3±0.210 | **2.36±0.049** | **1.22±0.092** | **-1.14±2.101** |
| Residential areas weighted % ±SE |  |  |  |  |  |  |  |  |  |  |  |  |
| Urban | 50.7±0.141 | 51.5±0.142 | 53.5±0.141 | 55.3±0.141 | 55.9±0.151 | 62.4±0.149 | 60.8±0.150 | 60.9±0.149 | 62.4±0.145 | **2.07±0.035** | 0.03±0.066 | **-2.04±3.030** |
| Rural | 35.8±0.161 | 37.9±0.161 | 39.6±0.162 | 39.6±0.161 | 43.8±0.193 | 47.6±0.185 | 47.8±0.186 | 48.0±0.188 | 50.2±0.186 | **2.15±0.041** | **0.80±0.083** | **-1.35±2.227** |
| Household income weighted % ±SE |  |  |  |  |  |  |  |  |  |  |  |  |
| Lowest quartile | 20.1±0.193 | 21.9±0.201 | 22.4±0.208 | 22.2±0.206 | 24.2±0.273 | 27.0±0.266 | 28.4±0.266 | 29.7±0.277 | 28.8±0.280 | **1.14±0.055** | **0.69±0.122** | **-0.44±1.173** |
| Second quartile | 43.5±0.177 | 43.6±0.178 | 45.5±0.181 | 45.1±0.184 | 45.1±0.213 | 50.9±0.212 | 50.9±0.209 | 50.5±0.212 | 51.4±0.213 | **1.14±0.046** | 0.12±0.095 | **-1.02±1.354** |
| Third quartile | 55.7±0.204 | 56.5±0.202 | 57.6±0.200 | 59.0±0.199 | 59.7±0.226 | 65.4±0.223 | 64.3±0.228 | 63.9±0.231 | 65.0±0.228 | **1.65±0.051** | -0.16±0.101 | **-1.81±2.186** |
| Highest quartile | 58.4±0.265 | 60.8±0.261 | 62.6±0.247 | 64.8±0.231 | 64.0±0.219 | 70.6±0.204 | 69.1±0.210 | 68.6±0.205 | 70.7±0.188 | **2.11±0.056** | 0.01±0.088 | **-2.10±2.479** |
| Educational level weighted % ±SE |  |  |  |  |  |  |  |  |  |  |  |  |
| Elementary school or less | 13.2±0.156 | 15.4±0.166 | 16.4±0.171 | 16.7±0.171 | 20.8±0.214 | 25.1±0.209 | 24.0±0.216 | 24.8±0.224 | 25.5±0.224 | **2.15±0.044** | 0.18±0.097 | **-1.97±2.490** |
| Middle school | 31.1±0.294 | 33.1±0.297 | 34.7±0.303 | 35.1±0.299 | 39.6±0.343 | 47.4±0.343 | 45.5±0.346 | 45.2±0.353 | 47.6±0.341 | **2.84±0.076** | 0.02±0.153 | **-2.82±3.724** |
| High school | 49.3±0.197 | 49.9±0.198 | 52.1±0.199 | 53.3±0.198 | 55.4±0.216 | 63.6±0.211 | 61.2±0.212 | 60.5±0.212 | 62.8±0.207 | **2.48±0.049** | **-0.29±0.094** | **-2.77±3.031** |
| College or more | 63.8±0.172 | 64.6±0.170 | 66.3±0.166 | 67.8±0.164 | 68.5±0.182 | 74.7±0.173 | 72.9±0.174 | 72.2±0.172 | 73.9±0.166 | **1.89±0.041** | **-0.27±0.076** | **-2.16±2.473** |
| Smoking status weighted % ±SE |  |  |  |  |  |  |  |  |  |  |  |  |
| Non-smoker | 49.9±0.137 | 51.4±0.136 | 53.1±0.136 | 53.8±0.134 | 56.2±0.149 | 59.6±0.146 | 58.3±0.144 | 59.0±0.145 | 60.7±0.145 | **1.79±0.034** | **0.41±0.065** | **-1.38±1.694** |
| Ex-smoker | 32.8±0.246 | 34.1±0.236 | 35.7±0.237 | 36.9±0.239 | 39.4±0.272 | 48.4±0.264 | 47.9±0.278 | 47.8±0.273 | 51.0±0.251 | **2.73±0.061** | **0.82±0.116** | **-1.91±2.299** |
| Current smoker | 38.0±0.230 | 38.6±0.242 | 41.8±0.244 | 42.8±0.250 | 46.4±0.281 | 53.5±0.290 | 52.7±0.293 | 52.1±0.296 | 54.2±0.287 | **2.80±0.062** | 0.15±0.129 | **-2.65±3.552** |
| Alcohol consumption, days/month weighted % ±SE |  |  |  |  |  |  |  |  |  |  |  |  |
| <1 | 40.7±0.157 | 42.2±0.157 | 43.3±0.156 | 43.6±0.155 | 46.9±0.173 | 50.9±0.165 | 51.3±0.159 | 51.9±0.157 | 52.8±0.159 | **1.86±0.039** | **0.63±0.072** | **-1.23±1.270** |
| 1-4 | 53.9±0.192 | 54.6±0.192 | 57.0±0.191 | 58.9±0.191 | 60.2±0.214 | 66.5±0.208 | 65.1±0.218 | 65.2±0.223 | 67.3±0.206 | **2.30±0.048** | **0.25±0.093** | **-2.04±2.093** |
| ≥5 | 39.5±0.220 | 40.8±0.221 | 43.6±0.227 | 45.4±0.225 | 49.0±0.253 | 55.5±0.261 | 54.2±0.282 | 54.5±0.286 | 56.4±0.271 | **2.98±0.057** | **0.27±0.120** | **-2.71±2.774** |
| Subjective health level weighted % ±SE |  |  |  |  |  |  |  |  |  |  |  |  |
| High | 53.6±0.176 | 54.5±0.174 | 56.5±0.174 | 57.8±0.173 | 58.3±0.197 | 65.4±0.194 | 62.6±0.166 | 63.1±0.181 | 66.2±0.175 | **1.99±0.044** | **0.42±0.082** | **-1.57±1.615** |
| Normal | 46.0±0.164 | 47.1±0.166 | 49.3±0.164 | 50.5±0.164 | 53.2±0.178 | 58.9±0.173 | 54.5±0.190 | 56.9±0.180 | 59.1±0.179 | **2.38±0.041** | **0.23±0.079** | **-2.15±2.189** |
| Low | 24.1±0.208 | 25.9±0.213 | 27.2±0.217 | 27.7±0.214 | 33.7±0.259 | 36.6±0.246 | 33.6±0.303 | 36.2±0.274 | 36.1±0.257 | **2.43±0.055** | 0.01±0.114 | **-2.42±2.483** |

BMI, body mass index; CI, confidence interval; KCHS, Korea Community Health Survey; SE, standard error.

The beta values were multiplied by 100 as a result of their minimal number.

Numbers in bold indicate a significant difference (P < 0.05).

**Table S3.** Prevalence of food labeling comprehension in the KCHS with SE, 2014–2022 (n =1,756,847).

|  | **2014** | **2015** | **2016** | **2017** | **2018** | **2019** | **2020** | **2021** | **2022** | **before the pandemic, β (2014-2019)** | **after the pandemic, β (2019-2022)** | **Trend difference, βdiff** |
| --- | --- | --- | --- | --- | --- | --- | --- | --- | --- | --- | --- | --- |
| Overall weighted % ±SE | 21.7±0.089 | 21.2±0.088 | 21.8±0.089 | 22.0±0.089 | 21.1±0.098 | 23.5±0.100 | 24.0±0.101 | 25.3±0.103 | 25.7±0.102 | **0.26±0.022** | **0.79±0.045** | **0.54±0.000** |
| Age group, year weighted% ±SE |  |  |  |  |  |  |  |  |  |  |  |  |
| 19-39 | 35.3±0.197 | 33.9±0.200 | 35.2±0.203 | 35.5±0.208 | 33.8±0.231 | 36.9±0.246 | 37.3±0.242 | 37.6±0.246 | 38.8±0.252 | **0.19±0.052** | **0.60±0.111** | **0.41±1.044** |
| 40-49 | 30.0±0.221 | 29.9±0.226 | 31.0±0.231 | 32.8±0.239 | 30.3±0.268 | 35.2±0.289 | 35.2±0.290 | 36.1±0.296 | 36.5±0.297 | **0.80±0.060** | **0.47±0.131** | **-0.33±1.844** |
| 50-59 | 19.1±0.187 | 19.9±0.190 | 20.8±0.192 | 22.1±0.197 | 21.6±0.220 | 26.5±0.240 | 27.1±0.242 | 29.5±0.252 | 30.1±0.251 | **1.21±0.050** | **1.31±0.110** | 0.09±1.030 |
| 60-69 | 9.7±0.161 | 11.1±0.164 | 11.6±0.166 | 12.5±0.166 | 13.8±0.190 | 18.0±0.204 | 18.3±0.205 | 20.9±0.210 | 22.5±0.208 | **1.44±0.044** | **1.63±0.092** | 0.19±0.486 |
| ≥70 | 3.3±0.096 | 4.0±0.101 | 4.0±0.100 | 4.1±0.097 | 4.9±0.114 | 6.8±0.120 | 6.6±0.121 | 8.0±0.132 | 8.7±0.131 | **0.60±0.026** | **0.72±0.056** | **0.12±4.362** |
| Sex weighted % ±SE |  |  |  |  |  |  |  |  |  |  |  |  |
| Male | 12.4±0.104 | 12.1±0.103 | 13.0±0.106 | 13.1±0.107 | 12.5±0.117 | 15.3±0.127 | 16.4±0.131 | 17.6±0.134 | 18.1±0.134 | **0.44±0.027** | **0.96±0.058** | **0.53±3.002** |
| Female | 29.9±0.135 | 29.0±0.134 | 29.4±0.134 | 29.6±0.133 | 28.4±0.147 | 30.1±0.145 | 30.3±0.147 | 31.7±0.149 | 32.1±0.147 | -0.01±0.033 | **0.72±0.066** | **0.73±2.953** |
| BMI group weighted % ±SE |  |  |  |  |  |  |  |  |  |  |  |  |
| Underweight | 25.7±0.409 | 24.7±0.411 | 24.3±0.415 | 24.7±0.420 | 23.9±0.505 | 26.9±0.507 | 24.9±0.498 | 26.7±0.496 | 26.7±0.482 | 0.08±0.109 | 0.11±0.221 | 0.03±1.964 |
| Normal weight | 24.5±0.138 | 23.9±0.138 | 24.4±0.141 | 24.9±0.142 | 23.8±0.161 | 26.7±0.172 | 26.3±0.163 | 27.2±0.165 | 27.8±0.164 | **0.30±0.036** | **0.41±0.075** | 0.11±0.598 |
| Overweight | 19.1±0.172 | 18.6±0.168 | 19.4±0.172 | 19.4±0.170 | 18.9±0.189 | 21.3±0.199 | 22.1±0.198 | 23.5±0.203 | 23.9±0.200 | **0.33±0.044** | **0.91±0.089** | **0.58±0.631** |
| Obese | 18.2±0.166 | 18.4±0.164 | 19.3±0.163 | 19.4±0.162 | 19.0±0.167 | 21.2±0.163 | 22.4±0.180 | 23.9±0.185 | 24.4±0.183 | **0.50±0.039** | **1.12±0.078** | **0.62±0.346** |
| Residential areas weighted % ±SE |  |  |  |  |  |  |  |  |  |  |  |  |
| Urban | 25.6±0.123 | 25.2±0.123 | 25.8±0.124 | 26.3±0.125 | 24.5±0.131 | 27.6±0.137 | 28.0±0.138 | 29.0±0.139 | 29.3±0.137 | **0.23±0.031** | **0.59±0.061** | **0.36±0.635** |
| Rural | 16.1±0.123 | 15.7±0.121 | 16.3±0.122 | 16.2±0.121 | 15.6±0.141 | 17.5±0.141 | 18.2±0.144 | 19.6±0.150 | 20.4±0.150 | **0.20±0.031** | **0.98±0.065** | **0.78±0.107** |
| Household income weighted % ±SE |  |  |  |  |  |  |  |  |  |  |  |  |
| Lowest quartile | 8.1±0.132 | 8.2±0.134 | 8.3±0.138 | 8.0±0.135 | 7.5±0.168 | 9.0±0.171 | 9.9±0.176 | 11.3±0.191 | 10.3±0.187 | 0.05±0.036 | **0.53±0.080** | **0.48±0.272** |
| Second quartile | 20.4±0.144 | 19.3±0.142 | 19.8±0.145 | 19.0±0.145 | 16.7±0.160 | 19.3±0.167 | 20.3±0.169 | 21.3±0.173 | 21.4±0.175 | **-0.40±0.037** | **0.73±0.076** | **1.13±0.778** |
| Third quartile | 27.8±0.184 | 27.0±0.181 | 27.4±0.180 | 27.7±0.181 | 25.4±0.200 | 28.0±0.210 | 28.7±0.215 | 29.9±0.220 | 29.8±0.218 | **-0.10±0.047** | **0.65±0.096** | **0.76±0.373** |
| Highest quartile | 31.0±0.249 | 31.0±0.248 | 31.0±0.236 | 32.3±0.226 | 28.8±0.206 | 32.2±0.209 | 32.6±0.214 | 33.2±0.208 | 33.7±0.196 | 0.03±0.055 | **0.52±0.091** | **0.49±0.111** |
| Educational level weighted % ±SE |  |  |  |  |  |  |  |  |  |  |  |  |
| Elementary school or less | 3.9±0.089 | 4.2±0.092 | 4.2±0.093 | 4.3±0.093 | 4.8±0.112 | 6.5±0.119 | 5.8±0.119 | 6.7±0.130 | 6.7±0.129 | **0.42±0.025** | **0.16±0.055** | **-0.26±0.800** |
| Middle school | 11.2±0.200 | 11.4±0.201 | 12.2±0.208 | 11.9±0.203 | 12.2±0.230 | 15.6±0.249 | 15.3±0.250 | 16.3±0.262 | 17.5±0.259 | **0.67±0.053** | **0.67±0.114** | 0.00±0.923 |
| High school | 22.7±0.165 | 22.2±0.164 | 22.6±0.167 | 22.8±0.167 | 21.5±0.179 | 25.5±0.191 | 25.2±0.189 | 26.5±0.192 | 27.0±0.190 | **0.33±0.042** | **0.58±0.085** | **0.26±0.007** |
| College or more | 34.8±0.170 | 33.6±0.168 | 34.3±0.167 | 34.9±0.167 | 32.5±0.183 | 36.2±0.191 | 36.7±0.188 | 37.2±0.186 | 37.6±0.183 | **0.10±0.043** | **0.47±0.084** | **0.38±0.062** |
| Smoking status weighted % ±SE |  |  |  |  |  |  |  |  |  |  |  |  |
| Non-smoker | 27.6±0.122 | 27.0±0.121 | 27.5±0.122 | 27.5±0.120 | 26.2±0.132 | 28.4±0.134 | 28.2±0.132 | 29.7±0.134 | 30.4±0.136 | 0.05±0.030 | **0.75±0.060** | **0.70±0.384** |
| Ex-smoker | 11.0±0.164 | 11.1±0.157 | 11.7±0.159 | 11.8±0.160 | 11.2±0.176 | 14.8±0.187 | 15.7±0.202 | 16.7±0.204 | 18.1±0.193 | **0.56±0.041** | **1.11±0.086** | **0.55±0.143** |
| Current smoker | 12.5±0.156 | 11.8±0.161 | 13.0±0.166 | 13.3±0.172 | 13.0±0.190 | 15.2±0.209 | 16.6±0.218 | 17.5±0.225 | 18.1±0.222 | **0.48±0.043** | **0.94±0.097** | **0.46±0.467** |
| Alcohol consumption, days/month weighted % ±SE |  |  |  |  |  |  |  |  |  |  |  |  |
| <1 | 21.6±0.131 | 21.1±0.130 | 21.3±0.129 | 20.9±0.127 | 20.6±0.140 | 22.2±0.138 | 23.1±0.134 | 24.7±0.136 | 24.7±0.138 | 0.04±0.032 | **0.91±0.061** | **0.87±0.833** |
| 1-4 | 27.0±0.171 | 26.0±0.169 | 27.0±0.171 | 27.7±0.174 | 25.6±0.191 | 29.0±0.200 | 29.2±0.208 | 30.0±0.214 | 31.2±0.204 | **0.28±0.044** | **0.73±0.091** | **0.46±0.406** |
| ≥5 | 14.7±0.159 | 14.7±0.159 | 15.6±0.166 | 16.8±0.169 | 16.2±0.186 | 18.9±0.205 | 19.3±0.223 | 20.2±0.230 | 20.4±0.220 | **0.76±0.043** | **0.52±0.096** | **-0.25±0.297** |
| Subjective health level weighted % ±SE |  |  |  |  |  |  |  |  |  |  |  |  |
| High | 27.0±0.156 | 26.2±0.153 | 27.0±0.156 | 27.6±0.157 | 25.1±0.173 | 29.1±0.185 | 28.8±0.155 | 30.4±0.172 | 31.7±0.172 | **0.22±0.040** | **0.97±0.079** | **0.75±0.710** |
| Normal | 22.4±0.138 | 21.7±0.137 | 22.4±0.137 | 22.6±0.138 | 21.7±0.147 | 24.4±0.151 | 22.6±0.160 | 25.1±0.158 | 25.8±0.159 | **0.28±0.034** | **0.66±0.070** | **0.38±0.342** |
| Low | 10.0±0.146 | 10.2±0.147 | 10.5±0.149 | 10.5±0.147 | 12.1±0.179 | 12.9±0.171 | 11.4±0.203 | 14.1±0.199 | 13.1±0.180 | **0.56±0.038** | **0.28±0.079** | **-0.28±0.328** |

BMI, body mass index; CI, confidence interval; KCHS, Korea Community Health Survey; SE, standard error.

The beta values were multiplied by 100 as a result of their minimal number.

Numbers in bold indicate a significant difference (P < 0.05).

**Table S4.** Prevalence of food labeling usage in the KCHS with SE, 2014–2022 (n =1,756,847).

|  | **2014** | **2015** | **2016** | **2017** | **2018** | **2019** | **2020** | **2021** | **2022** | **before the pandemic, β (2014-2019)** | **after the pandemic, β (2019-2022)** | **Trend difference, βdiff** |
| --- | --- | --- | --- | --- | --- | --- | --- | --- | --- | --- | --- | --- |
| Overall weighted % ±SE | 17.6±0.082 | 17.0±0.081 | 17.6±0.082 | 17.7±0.082 | 16.9±0.090 | 18.8±0.092 | 19.7±0.094 | 21.0±0.097 | 21.3±0.096 | **0.17±0.021** | **0.87±0.042** | **0.69±0.156** |
| Age group, year weighted% ±SE |  |  |  |  |  |  |  |  |  |  |  |  |
| 19-39 | 28.9±0.187 | 27.5±0.189 | 28.6±0.192 | 28.7±0.196 | 27.4±0.218 | 30.1±0.234 | 31.0±0.232 | 31.4±0.236 | 32.7±0.243 | **0.12±0.050** | **0.82±0.106** | **0.69±0.764** |
| 40-49 | 24.7±0.208 | 24.8±0.213 | 25.4±0.218 | 27.1±0.226 | 24.9±0.253 | 28.9±0.274 | 29.5±0.277 | 30.9±0.285 | 31.1±0.286 | **0.64±0.057** | **0.80±0.125** | **0.16±1.354** |
| 50-59 | 15.3±0.171 | 15.7±0.173 | 16.7±0.177 | 17.5±0.181 | 17.2±0.202 | 21.3±0.222 | 22.4±0.227 | 24.6±0.238 | 25.1±0.237 | **0.98±0.046** | **1.36±0.103** | **0.38±0.749** |
| 60-69 | 7.2±0.141 | 8.4±0.145 | 9.0±0.148 | 9.7±0.149 | 10.6±0.170 | 13.6±0.182 | 14.5±0.187 | 17.0±0.194 | 18.0±0.191 | **1.13±0.039** | **1.55±0.084** | **0.42±0.256** |
| ≥70 | 2.4±0.082 | 2.8±0.086 | 2.9±0.085 | 3.0±0.083 | 3.5±0.097 | 5.1±0.104 | 5.0±0.107 | 6.1±0.117 | 6.6±0.116 | **0.46±0.023** | **0.58±0.049** | **0.12±4.366** |
| Sex weighted % ±SE |  |  |  |  |  |  |  |  |  |  |  |  |
| Male | 9.3±0.092 | 9.2±0.091 | 9.7±0.094 | 9.8±0.094 | 9.2±0.102 | 11.5±0.113 | 12.9±0.118 | 14.0±0.123 | 14.4±0.122 | **0.30±0.024** | **1.01±0.053** | **0.71±2.824** |
| Female | 24.8±0.128 | 23.9±0.126 | 24.4±0.126 | 24.5±0.126 | 23.4±0.138 | 24.8±0.137 | 25.4±0.139 | 26.8±0.142 | 27.0±0.140 | -0.04±0.032 | **0.81±0.062** | **0.85±2.838** |
| BMI group weighted % ±SE |  |  |  |  |  |  |  |  |  |  |  |  |
| Underweight | 21.6±0.385 | 20.0±0.382 | 19.8±0.385 | 20.3±0.392 | 19.6±0.471 | 22.2±0.475 | 20.8±0.468 | 22.5±0.468 | 22.4±0.455 | 0.02±0.102 | 0.23±0.208 | 0.21±1.796 |
| Normal weight | 20.2±0.129 | 19.5±0.129 | 20.1±0.131 | 20.4±0.132 | 19.5±0.150 | 22.0±0.161 | 22.1±0.154 | 23.0±0.156 | 23.4±0.155 | **0.24±0.034** | **0.54±0.070** | **0.29±0.411** |
| Overweight | 15.4±0.158 | 14.8±0.154 | 15.5±0.157 | 15.5±0.155 | 15.0±0.173 | 16.9±0.182 | 18.0±0.183 | 19.4±0.189 | 19.6±0.186 | **0.23±0.040** | **0.94±0.082** | **0.70±0.508** |
| Obese | 14.1±0.150 | 14.3±0.148 | 15.0±0.148 | 15.1±0.147 | 14.7±0.151 | 16.4±0.148 | 17.8±0.165 | 19.4±0.172 | 19.7±0.169 | **0.38±0.036** | **1.14±0.071** | **0.76±0.209** |
| Residential areas weighted % ±SE |  |  |  |  |  |  |  |  |  |  |  |  |
| Urban | 20.9±0.115 | 20.4±0.114 | 21.1±0.116 | 21.5±0.116 | 19.9±0.121 | 22.4±0.128 | 23.2±0.129 | 24.3±0.131 | 24.5±0.129 | **0.17±0.029** | **0.76±0.058** | **0.59±0.405** |
| Rural | 12.8±0.112 | 12.3±0.109 | 12.8±0.111 | 12.6±0.109 | 12.0±0.126 | 13.7±0.127 | 14.7±0.132 | 16.0±0.138 | 16.4±0.138 | **0.10±0.028** | **0.93±0.059** | **0.83±0.062** |
| Household income weighted % ±SE |  |  |  |  |  |  |  |  |  |  |  |  |
| Lowest quartile | 6.2±0.117 | 6.2±0.117 | 6.2±0.120 | 6.1±0.119 | 5.5±0.145 | 6.7±0.149 | 7.7±0.157 | 8.8±0.172 | 7.8±0.166 | 0.00±0.031 | **0.48±0.071** | **0.48±0.282** |
| Second quartile | 16.0±0.131 | 15.2±0.129 | 15.6±0.132 | 14.9±0.132 | 12.9±0.143 | 15.2±0.152 | 16.2±0.154 | 17.2±0.160 | 17.1±0.161 | **-0.34±0.033** | **0.68±0.070** | **1.02±0.670** |
| Third quartile | 22.8±0.172 | 21.9±0.168 | 22.3±0.168 | 22.3±0.168 | 20.5±0.186 | 22.4±0.195 | 23.5±0.201 | 24.9±0.208 | 24.9±0.206 | **-0.18±0.043** | **0.89±0.090** | **1.06±0.674** |
| Highest quartile | 26.1±0.237 | 25.9±0.235 | 25.9±0.223 | 27.0±0.214 | 23.7±0.194 | 26.5±0.198 | 27.6±0.204 | 28.3±0.199 | 28.7±0.187 | **-0.11±0.052** | **0.71±0.086** | **0.82±0.431** |
| Educational level weighted % ±SE |  |  |  |  |  |  |  |  |  |  |  |  |
| Elementary school or less | 2.8±0.076 | 3.0±0.078 | 3.0±0.079 | 3.1±0.079 | 3.3±0.094 | 4.6±0.101 | 4.2±0.102 | 5.0±0.113 | 4.8±0.109 | **0.28±0.021** | **0.13±0.047** | **-0.15±0.697** |
| Middle school | 8.1±0.174 | 8.4±0.175 | 9.0±0.183 | 8.8±0.177 | 8.9±0.200 | 11.8±0.221 | 11.5±0.221 | 12.6±0.235 | 13.1±0.231 | **0.55±0.046** | **0.52±0.101** | -0.02±0.958 |
| High school | 17.8±0.151 | 17.2±0.149 | 17.6±0.152 | 17.8±0.152 | 16.7±0.162 | 19.8±0.175 | 20.1±0.174 | 21.4±0.178 | 21.7±0.177 | **0.23±0.038** | **0.72±0.079** | **0.50±0.228** |
| College or more | 29.2±0.162 | 28.1±0.160 | 28.7±0.159 | 29.0±0.159 | 27.0±0.174 | 30.2±0.183 | 31.3±0.181 | 31.9±0.179 | 32.5±0.177 | 0.05±0.041 | **0.74±0.081** | **0.69±0.373** |
| Smoking status weighted % ±SE |  |  |  |  |  |  |  |  |  |  |  |  |
| Non-smoker | 22.8±0.115 | 22.1±0.113 | 22.7±0.114 | 22.6±0.113 | 21.5±0.123 | 23.3±0.125 | 23.5±0.124 | 25.1±0.128 | 25.6±0.129 | 0.01±0.029 | **0.85±0.057** | **0.84±0.525** |
| Ex-smoker | 8.5±0.146 | 8.5±0.139 | 8.9±0.141 | 9.1±0.143 | 8.6±0.156 | 11.2±0.167 | 12.5±0.184 | 13.4±0.187 | 14.7±0.177 | **0.41±0.037** | **1.12±0.078** | **0.71±0.291** |
| Current smoker | 9.1±0.136 | 8.7±0.140 | 9.5±0.145 | 9.5±0.149 | 9.2±0.163 | 11.1±0.182 | 12.5±0.194 | 13.3±0.201 | 13.9±0.200 | **0.30±0.037** | **0.93±0.086** | **0.63±0.300** |
| Alcohol consumption, days/month weighted % ±SE |  |  |  |  |  |  |  |  |  |  |  |  |
| <1 | 17.8±0.122 | 17.2±0.120 | 17.5±0.120 | 17.0±0.117 | 16.7±0.129 | 18.0±0.127 | 19.1±0.125 | 20.7±0.127 | 20.5±0.129 | -0.03±0.030 | **0.92±0.057** | **0.95±0.916** |
| 1-4 | 21.8±0.159 | 21.0±0.157 | 21.7±0.159 | 22.3±0.162 | 20.6±0.177 | 23.4±0.187 | 24.0±0.196 | 24.9±0.202 | 26.1±0.193 | **0.21±0.041** | **0.89±0.085** | **0.68±0.634** |
| ≥5 | 11.2±0.142 | 11.2±0.142 | 12.0±0.148 | 13.0±0.152 | 12.4±0.167 | 14.5±0.185 | 15.2±0.203 | 16.3±0.212 | 16.3±0.201 | **0.59±0.038** | **0.62±0.087** | 0.03±0.017 |
| Subjective health level weighted % ±SE |  |  |  |  |  |  |  |  |  |  |  |  |
| High | 22.1±0.146 | 21.3±0.143 | 21.9±0.146 | 22.5±0.146 | 20.5±0.161 | 23.6±0.173 | 23.9±0.146 | 25.6±0.163 | 26.7±0.164 | **0.15±0.037** | **1.12±0.074** | **0.96±0.922** |
| Normal | 18.0±0.127 | 17.4±0.126 | 18.0±0.126 | 18.1±0.127 | 17.1±0.134 | 19.4±0.139 | 18.4±0.148 | 20.7±0.147 | 21.1±0.148 | **0.18±0.032** | **0.72±0.065** | **0.54±0.501** |
| Low | 7.8±0.131 | 8.0±0.132 | 8.3±0.134 | 8.1±0.130 | 9.5±0.161 | 10.0±0.154 | 9.0±0.184 | 11.3±0.181 | 10.4±0.164 | **0.44±0.034** | **0.30±0.072** | -0.14±0.177 |

BMI, body mass index; CI, confidence interval; KCHS, Korea Community Health Survey; SE, standard error.

The beta values were multiplied by 100 as a result of their minimal number.

Numbers in bold indicate a significant difference (P < 0.05).

**Table S5.** Adjusted odds ratios of food labeling awareness among pre-pandemic and during the pandemic, ORs (95% CI), in the data gathered from the KCHS.

|  | **Adjusted odds ratio (OR) before and after the COVID-19 pandemic  (2020 vs 2019)** | **Adjusted odds ratio (OR) before and after the COVID-19 pandemic  (2021 vs 2020)** | **Adjusted odds ratio (OR) before and after the COVID-19 pandemic  (2022 vs 2021)** |
| --- | --- | --- | --- |
| Overall | **0.900 (0.887 to 0.914)** | 1.013 (0.998 to 1.028) | **1.111 (1.095 to 1.128)** |
| 19-39 | **0.910 (0.880 to 0.941)** | **0.960 (0.929 to 0.992)** | **1.123 (1.086 to 1.161)** |
| 40-49 | **0.865 (0.831 to 0.900)** | 0.970 (0.933 to 1.009) | **1.046 (1.006 to 1.088)** |
| 50-59 | **0.899 (0.869 to 0.929)** | 0.987 (0.955 to 1.020) | **1.121 (1.084 to 1.159)** |
| 60-69 | **0.913 (0.885 to 0.942)** | **1.058 (1.026 to 1.090)** | **1.124 (1.092 to 1.158)** |
| ≥70 | **0.890 (0.860 to 0.920)** | **1.101 (1.065 to 1.138)** | **1.143 (1.107 to 1.180)** |
| Male | **0.924 (0.904 to 0.944)** | 0.995 (0.975 to 1.017) | **1.118 (1.095 to 1.142)** |
| Female | **0.876 (0.858 to 0.895)** | **1.030 (1.009 to 1.052)** | **1.106 (1.083 to 1.129)** |
| Underweight | **0.826 (0.764 to 0.893)** | 1.013 (0.939 to 1.093) | **1.156 (1.073 to 1.245)** |
| Normal weight | **0.890 (0.868 to 0.912)** | 0.999 (0.976 to 1.023) | **1.117 (1.091 to 1.143)** |
| Overweight | **0.904 (0.877 to 0.931)** | **1.030 (1.001 to 1.061)** | **1.112 (1.080 to 1.145)** |
| Obese | **0.917 (0.894 to 0.941)** | 1.018 (0.992 to 1.045) | **1.100 (1.072 to 1.129)** |
| Urban | **0.888 (0.871 to 0.905)** | 1.018 (0.999 to 1.038) | **1.105 (1.084 to 1.126)** |
| Rural | **0.915 (0.893 to 0.937)** | 1.003 (0.980 to 1.027) | **1.124 (1.098 to 1.151)** |
| Lowest quartile | **0.887 (0.851 to 0.925)** | 1.103 (1.059 to 1.149) | 1.027 (0.985 to 1.071) |
| Second quartile | **0.902 (0.879 to 0.926)** | 1.021 (0.996 to 1.048) | **1.121 (1.093 to 1.150)** |
| Third quartile | **0.912 (0.886 to 0.940)** | 0.992 (0.963 to 1.021) | **1.112 (1.080 to 1.145)** |
| Highest quartile | **0.878 (0.852 to 0.903)** | 0.980 (0.953 to 1.008) | **1.142 (1.112 to 1.173)** |
| Elementary school or less | **0.911 (0.880 to 0.943)** | **1.123 (1.084 to 1.163)** | **1.099 (1.061 to 1.138)** |
| Middle school | **0.909 (0.872 to 0.947)** | 1.033 (0.991 to 1.076) | **1.140 (1.095 to 1.187)** |
| High school | **0.890 (0.866 to 0.914)** | 0.998 (0.973 to 1.024) | **1.131 (1.103 to 1.161)** |
| College or more | **0.887 (0.865 to 0.911)** | 0.977 (0.953 to 1.002) | **1.107 (1.080 to 1.134)** |
| Non-smoker | **0.880 (0.863 to 0.897)** | **1.027 (1.008 to 1.046)** | **1.102 (1.081 to 1.123)** |
| Ex-smoker | **0.914 (0.884 to 0.945)** | 0.994 (0.962 to 1.028) | **1.150 (1.114 to 1.187)** |
| Current smoker | **0.948 (0.916 to 0.981)** | 0.987 (0.954 to 1.022) | **1.106 (1.068 to 1.144)** |
| <1 | **0.900 (0.881 to 0.919)** | **1.023 (1.003 to 1.044)** | **1.108 (1.086 to 1.131)** |
| 1-4 | **0.891 (0.865 to 0.916)** | 0.999 (0.971 to 1.028) | **1.125 (1.094 to 1.157)** |
| ≥5 | **0.916 (0.886 to 0.947)** | 1.004 (0.970 to 1.039) | **1.101 (1.065 to 1.139)** |
| High | **0.910 (0.889 to 0.932)** | 0.985 (0.964 to 1.007) | **1.130 (1.104 to 1.157)** |
| Normal | **0.889 (0.869 to 0.909)** | **1.031 (1.008 to 1.055)** | **1.115 (1.090 to 1.140)** |
| Low | **0.897 (0.863 to 0.932)** | **1.051 (1.010 to 1.094)** | **1.083 (1.044 to 1.123)** |

Abbreviations: CI, confidence interval; KCHS, Korea Community Health Survey; OR, odds ratio.

The beta values were multiplied by 100 as a result of their minimal number.

The numbers in bold indicate a significant difference (p < 0.05).

All models were adjusted for age (19 to 39, 40 to 49, 50 to 59, 60 to 69, and ≥70 years), sex, body mass index (BMI; underweight, normal weight, overweight, and obese), residential areas (urban and rural), household income (lowest quartile, second quartile, third quartile, and highest quartile), educational level (elementary school or less, middle school, high school, and college or more), smoking status (non-smoker, ex-smoker, and current smoker), alcohol consumption (below a day, once to four days, and five days or more per month), and subjective health level (high, normal, and low).

**Table S6.** Adjusted odds ratios of food labeling comprehension among pre-pandemic and during the pandemic, ORs (95% CI), in the data gathered from the KCHS.

|  | **Adjusted odds ratio (OR) before and after the COVID-19 pandemic  (2020 vs 2019)** | **Adjusted odds ratio (OR) before and after the COVID-19 pandemic  (2021 vs 2020)** | **Adjusted odds ratio (OR) before and after the COVID-19 pandemic  (2022 vs 2021)** |
| --- | --- | --- | --- |
| Overall | **0.964 (0.948 to 0.980)** | **1.082 (1.064 to 1.100)** | **1.050 (1.033 to 1.067)** |
| 19-39 | 0.991 (0.962 to 1.022) | 1.016 (0.986 to 1.046) | **1.053 (1.022 to 1.085)** |
| 40-49 | 0.967 (0.931 to 1.004) | 1.036 (0.998 to 1.075) | 1.001 (0.964 to 1.039) |
| 50-59 | 0.977 (0.942 to 1.013) | **1.108 (1.069 to 1.148)** | 1.031 (0.995 to 1.068) |
| 60-69 | **0.952 (0.914 to 0.991)** | **1.151 (1.107 to 1.196)** | **1.083 (1.044 to 1.123)** |
| ≥70 | **0.862 (0.815 to 0.913)** | **1.260 (1.194 to 1.331)** | **1.103 (1.049 to 1.159)** |
| Male | **1.037 (1.008 to 1.066)** | **1.102 (1.072 to 1.132)** | **1.071 (1.043 to 1.100)** |
| Female | **0.926 (0.907 to 0.946)** | **1.067 (1.045 to 1.090)** | **1.028 (1.007 to 1.049)** |
| Underweight | **0.894 (0.825 to 0.970)** | **1.095 (1.011 to 1.186)** | 1.006 (0.932 to 1.086) |
| Normal weight | **0.954 (0.929 to 0.980)** | **1.059 (1.032 to 1.086)** | **1.053 (1.027 to 1.079)** |
| Overweight | 0.997 (0.962 to 1.032) | **1.099 (1.062 to 1.137)** | **1.059 (1.024 to 1.095)** |
| Obese | **0.951 (0.923 to 0.980)** | **1.100 (1.067 to 1.134)** | **1.042 (1.011 to 1.073)** |
| Urban | **0.967 (0.947 to 0.987)** | **1.069 (1.048 to 1.091)** | **1.042 (1.022 to 1.063)** |
| Rural | **0.957 (0.929 to 0.986)** | **1.107 (1.075 to 1.139)** | **1.068 (1.038 to 1.098)** |
| Lowest quartile | **0.895 (0.841 to 0.952)** | **1.190 (1.123 to 1.262)** | 0.971 (0.915 to 1.030) |
| Second quartile | **0.956 (0.926 to 0.987)** | **1.110 (1.076 to 1.145)** | **1.074 (1.042 to 1.108)** |
| Third quartile | 0.986 (0.955 to 1.017) | **1.077 (1.044 to 1.111)** | **1.049 (1.017 to 1.081)** |
| Highest quartile | **0.963 (0.935 to 0.991)** | **1.039 (1.010 to 1.068)** | **1.053 (1.025 to 1.081)** |
| Elementary school or less | **0.840 (0.791 to 0.891)** | **1.283 (1.208 to 1.364)** | 1.059 (0.998 to 1.123) |
| Middle school | **0.937 (0.887 to 0.991)** | **1.137 (1.076 to 1.202)** | **1.135 (1.076 to 1.198)** |
| High school | **0.960 (0.932 to 0.989)** | **1.110 (1.078 to 1.142)** | **1.058 (1.028 to 1.088)** |
| College or more | 0.988 (0.964 to 1.012) | **1.034 (1.010 to 1.058)** | **1.037 (1.013 to 1.060)** |
| Non-smoker | **0.937 (0.918 to 0.956)** | **1.078 (1.058 to 1.099)** | **1.040 (1.020 to 1.060)** |
| Ex-smoker | 0.984 (0.941 to 1.029) | **1.089 (1.042 to 1.138)** | **1.093 (1.050 to 1.139)** |
| Current smoker | **1.086 (1.037 to 1.137)** | **1.093 (1.045 to 1.143)** | **1.047 (1.002 to 1.094)** |
| <1 | **0.945 (0.922 to 0.968)** | **1.105 (1.081 to 1.130)** | **1.051 (1.028 to 1.074)** |
| 1-4 | 0.979 (0.951 to 1.008) | **1.054 (1.023 to 1.085)** | **1.060 (1.030 to 1.091)** |
| ≥5 | 0.994 (0.954 to 1.036) | **1.061 (1.017 to 1.107)** | 1.027 (0.986 to 1.070) |
| High | 0.996 (0.972 to 1.021) | **1.059 (1.035 to 1.084)** | **1.056 (1.032 to 1.082)** |
| Normal | **0.948 (0.924 to 0.973)** | **1.087 (1.059 to 1.115)** | **1.061 (1.035 to 1.088)** |
| Low | **0.872 (0.825 to 0.921)** | **1.194 (1.129 to 1.263)** | 1.025 (0.976 to 1.077) |

Abbreviations: CI, confidence interval; KCHS, Korea Community Health Survey; OR, odds ratio.

The beta values were multiplied by 100 as a result of their minimal number.

The numbers in bold indicate a significant difference (p < 0.05).

All models were adjusted for age (19 to 39, 40 to 49, 50 to 59, 60 to 69, and ≥70 years), sex, body mass index (BMI; underweight, normal weight, overweight, and obese), residential areas (urban and rural), household income (lowest quartile, second quartile, third quartile, and highest quartile), educational level (elementary school or less, middle school, high school, and college or more), smoking status (non-smoker, ex-smoker, and current smoker), alcohol consumption (below a day, once to four days, and five days or more per month), and subjective health level (high, normal, and low).

**Table S7.** Adjusted odds ratios of food labeling usage among pre-pandemic and during the pandemic, ORs (95% CI), in the data gathered from the KCHS.

|  | **Adjusted odds ratio (OR) before and after the COVID-19 pandemic  (2020 vs 2019)** | **Adjusted odds ratio (OR) before and after the COVID-19 pandemic  (2021 vs 2020)** | **Adjusted odds ratio (OR) before and after the COVID-19 pandemic  (2022 vs 2021)** |
| --- | --- | --- | --- |
| Overall | 0.995 (0.977 to 1.013) | **1.091 (1.072 to 1.110)** | **1.041 (1.023 to 1.059)** |
| 19-39 | 1.015 (0.984 to 1.048) | 1.021 (0.990 to 1.053) | **1.061 (1.028 to 1.095)** |
| 40-49 | 0.995 (0.957 to 1.036) | **1.066 (1.025 to 1.108)** | 0.989 (0.951 to 1.028) |
| 50-59 | 1.011 (0.973 to 1.051) | **1.107 (1.066 to 1.150)** | 1.030 (0.993 to 1.069) |
| 60-69 | 0.995 (0.952 to 1.041) | **1.177 (1.128 to 1.227)** | **1.051 (1.010 to 1.093)** |
| ≥70 | **0.878 (0.823 to 0.936)** | **1.261 (1.186 to 1.341)** | **1.081 (1.022 to 1.144)** |
| Male | **1.080 (1.047 to 1.115)** | **1.122 (1.089 to 1.156)** | **1.067 (1.037 to 1.098)** |
| Female | **0.956 (0.935 to 0.977)** | **1.071 (1.048 to 1.095)** | **1.017 (0.995 to 1.039)** |
| Underweight | **0.908 (0.834 to 0.989)** | **1.088 (1.000 to 1.183)** | 1.000 (0.923 to 1.083) |
| Normal weight | 0.988 (0.961 to 1.016) | **1.061 (1.033 to 1.090)** | **1.048 (1.021 to 1.076)** |
| Overweight | 1.025 (0.987 to 1.065) | **1.113 (1.073 to 1.154)** | **1.043 (1.007 to 1.080)** |
| Obese | 0.980 (0.949 to 1.013) | **1.120 (1.084 to 1.157)** | 1.031 (0.999 to 1.064) |
| Urban | 0.997 (0.975 to 1.019) | **1.078 (1.055 to 1.101)** | **1.041 (1.020 to 1.063)** |
| Rural | 0.990 (0.959 to 1.022) | **1.118 (1.084 to 1.154)** | **1.042 (1.011 to 1.074)** |
| Lowest quartile | 0.940 (0.876 to 1.007) | **1.193 (1.118 to 1.273)** | 0.945 (0.884 to 1.009) |
| Second quartile | 0.973 (0.940 to 1.007) | **1.119 (1.082 to 1.157)** | **1.056 (1.022 to 1.092)** |
| Third quartile | 1.016 (0.983 to 1.051) | **1.099 (1.064 to 1.136)** | **1.047 (1.014 to 1.082)** |
| Highest quartile | 1.001 (0.971 to 1.032) | **1.043 (1.013 to 1.074)** | **1.047 (1.018 to 1.076)** |
| Elementary school or less | **0.866 (0.808 to 0.929)** | **1.306 (1.217 to 1.400)** | 1.009 (0.942 to 1.080) |
| Middle school | **0.929 (0.873 to 0.988)** | **1.176 (1.106 to 1.251)** | **1.088 (1.025 to 1.155)** |
| High school | 0.998 (0.966 to 1.030) | **1.124 (1.089 to 1.159)** | **1.045 (1.014 to 1.077)** |
| College or more | 1.017 (0.992 to 1.043) | **1.042 (1.017 to 1.067)** | **1.042 (1.018 to 1.067)** |
| Non-smoker | **0.965 (0.945 to 0.986)** | **1.089 (1.067 to 1.111)** | **1.027 (1.006 to 1.047)** |
| Ex-smoker | 1.035 (0.985 to 1.088) | **1.094 (1.042 to 1.148)** | **1.089 (1.042 to 1.139)** |
| Current smoker | **1.126 (1.069 to 1.186)** | **1.099 (1.045 to 1.155)** | **1.058 (1.008 to 1.111)** |
| <1 | **0.972 (0.948 to 0.997)** | **1.117 (1.090 to 1.144)** | **1.034 (1.010 to 1.059)** |
| 1-4 | 1.013 (0.981 to 1.045) | **1.052 (1.020 to 1.086)** | **1.067 (1.035 to 1.099)** |
| ≥5 | 1.030 (0.984 to 1.078) | **1.082 (1.033 to 1.133)** | 1.013 (0.969 to 1.059) |
| High | **1.030 (1.003 to 1.057)** | **1.072 (1.046 to 1.098)** | **1.053 (1.027 to 1.079)** |
| Normal | 0.975 (0.948 to 1.003) | **1.096 (1.066 to 1.127)** | **1.041 (1.014 to 1.069)** |
| Low | **0.895 (0.843 to 0.950)** | **1.183 (1.113 to 1.258)** | 1.028 (0.974 to 1.085) |

Abbreviations: CI, confidence interval; KCHS, Korea Community Health Survey; OR, odds ratio.

The beta values were multiplied by 100 as a result of their minimal number.

The numbers in bold indicate a significant difference (p < 0.05).

All models were adjusted for age (19 to 39, 40 to 49, 50 to 59, 60 to 69, and ≥70 years), sex, body mass index (BMI; underweight, normal weight, overweight, and obese), residential areas (urban and rural), household income (lowest quartile, second quartile, third quartile, and highest quartile), educational level (elementary school or less, middle school, high school, and college or more), smoking status (non-smoker, ex-smoker, and current smoker), alcohol consumption (below a day, once to four days, and five days or more per month), and subjective health level (high, normal, and low).
